# Role of Interaction and Nucleoside Diphosphate Kinase B in Regulation of the Cystic Fibrosis Transmembrane Conductance Regulator Function by cAMP-Dependent Protein Kinase A

**DOI:** 10.1371/journal.pone.0149097

**Published:** 2016-03-07

**Authors:** Lee A. Borthwick, Mathieu Kerbiriou, Christopher J. Taylor, Giorgio Cozza, Ioan Lascu, Edith H. Postel, Diane Cassidy, Pascal Trouvé, Anil Mehta, Louise Robson, Richmond Muimo

**Affiliations:** 1 Academic Unit of Respiratory Medicine, Department of Infection and Immunity, The University of Sheffield, The Medical School, Sheffield, S10 2RX, United Kingdom; 2 Academic Unit of Child Health, University of Sheffield, Stephenson Wing, Sheffield Children's Hospital, Sheffield, S10 2TH, United Kingdom; 3 Department of Biomedical Science, The University of Sheffield, Western Bank, Sheffield, S10 2TN, United Kingdom; 4 Department of Biomedical Sciences, University of Padova, Via Ugo Bassi 58/B 35131, Padova, Italy; 5 University of Bordeaux, France, and Institut de Biochimie et Genetique Cellulaires, Centre Nationale de la Recherche Scientifique UMR 5095, 33077, Bordeaux, France; 6 Robert Wood Johnson Medical School, UMDNJ, New Brunswick, New Jersey, United States of America; 7 Medical Research Institute/CVS Diabetes Lung, Ninewells Hospital and Medical School, University of Dundee, Dundee, DD1 9SY, United Kingdom; 8 Inserm UMR 1078, Génétique, Génomique Fonctionnelle et Biotechnologies, 46 rue Félix Le Dantec, 29218, Brest Cedex2, France; Indiana University, UNITED STATES

## Abstract

Cystic fibrosis results from mutations in the cystic fibrosis transmembrane conductance regulator (CFTR), a cAMP-dependent protein kinase A (PKA) and ATP-regulated chloride channel. Here, we demonstrate that nucleoside diphosphate kinase B (NDPK-B, NM23-H2) forms a functional complex with CFTR. In airway epithelia forskolin/IBMX significantly increases NDPK-B co-localisation with CFTR whereas PKA inhibitors attenuate complex formation. Furthermore, an NDPK-B derived peptide (but not its NDPK-A equivalent) disrupts the NDPK-B/CFTR complex *in vitro* (19-mers comprising amino acids 36–54 from NDPK-B or NDPK-A). Overlay (Far-Western) and Surface Plasmon Resonance (SPR) analysis both demonstrate that NDPK-B binds CFTR within its first nucleotide binding domain (NBD1, CFTR amino acids 351–727). Analysis of chloride currents reflective of CFTR or outwardly rectifying chloride channels (ORCC, DIDS-sensitive) showed that the 19-mer NDPK-B peptide (but not its NDPK-A equivalent) reduced both chloride conductances. Additionally, the NDPK-B (but not NDPK-A) peptide also attenuated acetylcholine-induced intestinal short circuit currents. *In silico* analysis of the NBD1/NDPK-B complex reveals an extended interaction surface between the two proteins. This binding zone is also target of the 19-mer NDPK-B peptide, thus confirming its capability to disrupt NDPK-B/CFTR complex. We propose that NDPK-B forms part of the complex that controls chloride currents in epithelia.

## Introduction

The importance of epithelial ion transport is highlighted by the disease cystic fibrosis (CF), a monogenic disorder resulting from mutations in the cystic fibrosis transmembrane conductance regulator (CFTR, ABCC7). CFTR is best characterized as a dual cAMP/PKA and ATP-regulated anion channel that is trafficked to the apical (luminal facing) membrane of polarized epithelia such as gut, airway and reproductive tract [1]. CFTR is also expressed in non-epithelial tissues such as lymphocytes and macrophages [2, 3] and this might explain why clinical CF disease manifests multiple cellular defects in addition to disrupted epithelial ion transport. These ‘non-channel functions’ include defective autophagy [4], unchecked inflammation that fails to resolve [5] and an excess of cancer [6, 7]. The pleiotropic effects of CFTR mutation are complex and no coherent model explains all aspects of the disease [8]. However, recent evidence show congenital abnormalities in various CF models suggesting defective airway development [9].

In 70–90% of CF patients, only one amino acid is deleted on one or both alleles to generate a F508del-CFTR mutant that folds inefficiently. F508del-CFTR is an ER-associated mutant that fails quality control and is not delivered to the plasma membrane [10], although there are data that disagree with this notion [11, 12]. Irrespective of this controversy, should some fraction of F508del-CFTR reach the plasma membrane, its residence time is shortened and the mutant (unlike wild type CFTR) additionally fails to recycle to the membrane [13]. It is established that CFTR does not act alone [8, 14] and recent evidence demonstrates functional roles for protein complexes bound to CFTR. For example, several transport-inhibitory proteins bind to CFTR, including syntaxin 1A and AMPKα [15, 16]. Correspondingly, reagents that disrupt such complexes potentiate CFTR function [17]. In addition, the appellation “regulator” in the naming of the CFTR channel describes the effect of CFTR mutation on the mis-control of other ion channels such as the outwardly rectifying chloride channel (ORCC) [18]. Hence, the signaling complexes and pathways that control CFTR are multiple and remain incompletely understood [8].

Nucleoside diphosphate kinases (NDPK, nm23, nme) belong to an eight member protein histidine kinase family divided into two groups (I and II). Only two closely homologous family members (NDPK-A & B, from group I) have been extensively investigated. As reviewed elsewhere, NDPK isoforms found in model systems that are similar to NDPK-A and -B control endocytosis [19, 20] and tracheal development [21]. These functions are in addition to the well established catalytic function of group 1 members in the synthesis of non-adenine nucleoside triphosphates [22–24]. The pleiotropic effects of this protein family on cellular processes including cell differentiation, growth and development, tumour metastasis and transcriptional processing are well established [25–27]. NDPK-A and—B share 88% sequence similarity and are thought to exist as heterohexamers in many cell types [28, 29]. Increasing evidence suggests that despite their highly homologous nature (their genes lie adjacent to one another), the cellular actions of NDPK-A & -B isoforms differ substantially. For example, NDPK-B (nme2, or nm23-H2), but not NDPK-A, binds and phosphorylates the G-protein β-subunit on a histidine residue (H226) thereby enhancing the basal activity of the G-protein α-subunit [30, 31]. Interestingly, regulation of the G protein-coupled receptor (thromboxane A2 receptor, TPβ) and the calcium dependent potassium channel (KCa3.1) is also specific to NDPK-B [32, 33]. In this regard, the protein histidine kinase acivity of NDPK-B regulates a calcium-activated potassium channel by direct transfer of its high energy phosphohistidine (on NDPK-B H118) to another histidine on the channel protein [33]. NDPK-B uses a similar ‘his-his’ energy transfer mechanism to promote the basal rate of cAMP production found in cells in the absence of G-protein coupled receptor occupancy [34]. The combined data suggest that distinct regulatory mechanisms control NDPK-A and -B function in vivo and that isoform NDPK-B is implicated in signaling events close to the plasma membrane of many different cell types.

We recently demonstrated a functional interaction between CFTR, AMPKα1 and NDPK-A, [35] which is independent of NDPK-B. These differences between NDPK-A and -B prompted our interest because heterotrimeric G-proteins regulate CFTR channel activity [36] and there exists sequence homology between G proteins and the region of the F508del-CFTR mutation in the first nucleotide binding domain of CFTR (NBD1) [37]. Furthermore, we have previously demonstrated that in epithelial membranes, NDPK histidine phosphorylation is itself regulated by both chloride and cation concentration *in vitro* [38–40]. Previous work has also shown that NDPK regulates the atrial muscarinic potassium channel but the mechanism is unknown [41]. Since cAMP not only plays a key role in CFTR-dependent chloride transport [42] but also regulates NDPK *in vitro* [43, 44], we investigated whether NDPK-B and CFTR might interact functionally in epithelia in a pathway involving cAMP and/or PKA (see discussion). We report that cAMP, acting through PKA regulates translocation of NDPK-B from the cytosol to the apical membrane leading to the formation of a functionally relevant complex between NDPK-B and CFTR. We demonstrate that the nucleotide binding domain 1 (NBD1, aa 351–727) of CFTR constitutes an NDPK-B interaction site with CFTR. Thus our data suggest that NDPK-B is important for the cAMP/PKA regulation of CFTR function.

## Materials and Methods

### Chemicals and reagents

All chemicals unless otherwise indicated were purchased from Sigma. PVDF membrane was purchased from Millipore (Watford, UK), acrylamide and other electrophoretic reagents were from BioRad (Hemel Hempsted, UK). N-[2-(p-bromocinnamylamino) ethyl]-5-isoquinolinesulfonamide (H-89), and myristoylated protein kinase A inhibitor amide 14–22 were purchased from Calbiochem (UK). Peptides (>95% purity) were obtained from Sigma Genosys (Dorset, UK). Fetal calf serum was purchased from Invitrogen Life Technologies (Renfrew, Strathclyde, UK).

### Cell culture

A wild type human bronchial epithelial cell line (16HBE14o-) [45]was grown in medium 199 containing fetal calf serum as described [46] until confluent. Membrane and cytosolic fractions were prepared as previously described [47].

### Ovine tracheal and human nasal epithelium (HNE)

Membrane and cytosolic fractions from ovine airway epithelia were prepared as described previously [47]. HNE were obtained as described before from healthy young adults undergoing surgery for reasons unrelated to nasal mucosal disease [48]. Local ethical committee (Tayside Committee on Medical Research Ethics) approval and written informed consent were obtained by AM (ref number 11/91). Nasal brushings were suspended in medium 199 until use or stored in liquid nitrogen.

### Expression and preparation of recombinant NBD1 domain

The CFTR fragment comprising the CFTR domains, nucleotide binding domain 1 (NBD1 domain—351 (TRQ>>) to 727 (GIEED)), R-domain -635 (NLQ>>) to 837 (FFDDM)) and nucleotide binding domain 2 (NBD2, 1151 (IDV>>) to 1360 (LARSV)) were amplified by PCR using human CFTR cDNA as template. The PCR product was then inserted into Xho1 restriction site of the bacterial pRSET-A plasmid (Invitrogen) carrying a 6xHis sequence upstream the multiple cloning site or pGEX4T-1 plasmid. The vectors were transformed into Escherichia coli strain BL21/DE3. The transformants were selected by ampicillin resistance (100 μg/mL) and the correct positive clones were screened by restriction digestion and their sequence checked by sequencing. Expression of recombinant proteins was induced by isopropyl β-D-thiogalactopyranoside (IPTG) (100 μM) for 2 hours at 25°C, 180 rpm. The induced cultures were centrifuged at 9,500 rpm for 20 minutes at 4°C and the bacterial pellet was then resuspended with lysis buffer (50 mM Tris-HCl, 100 mM NaCl, 10% glycerol, 0.1% NP-40, 1 mM EDTA, 0.1% Triton X-100 and proteases inhibitors). After sonication, the lysates were centrifuged 30 minutes at 9,500 rpm, 4°C and the supernatants added to a Ni-NTA (Qiagen, 30210)—Sepharose 4B (Sigma, CL4B200) beads mixture for the purification of the His-tagged proteins or Glutathione Sepharose beads for the purification of the GST-tagged proteins. The bound proteins were eluted with either Laemmli sample buffer (62.5 mM Tris-HCl, 25% glycerol, 2% SDS, 0.01% bromophenol blue, pH 6.8) plus 5% β-mercaptoethanol, (710 mM) for further far-western blot analysis or with HBS-EP buffer (10 mM HEPES, pH 7.4 containing 150 mM NaCl, 3 mM EDTA and 0.005% (v/v) Surfactant P20) for further Surface Plasmon Resonance (SPR) analysis. The quantity and purity of the domains was confirmed by Coomassie Blue staining of a 12% polyacrylamide gel.

### Gut biopsy and short-circuit current measurements

With local ethical committee approval and written informed consent (ref number—04/Q2305/83), a sheet of stripped intestine was obtained endoscopically from the distal ileum and the potential difference (PD), SCC and tissue resistance measured using a modified Ussing chamber technique as described previously [49]. Briefly, the sample was mounted in an Ussing chamber with an aperture of 0.03 cm^2^ and incubated at 37°C in Krebs bicarbonate saline gassed with 95% O_2_/5% CO_2_. The serosal fluid contained 10 mM glucose and the mucosal fluid 10 mM mannitol, each having a volume of 5 ml. The PD was measured using salt bridge electrodes connected via calomel half cells to a differential input electrometer with output to a two-channel chart recorder (Linseis L6512). Current was applied across the tissue via conductive plastic electrodes and tissue resistance determined from the PD change induced by a 50 μA current pulse, taking into account the fluid resistance. The SCC generated by the sheets was calculated from PD and resistance measurements using Ohm's law. The tissue was allowed to stabilize for 10 min after mounting and then readings of electrical activity taken at 1-min intervals. Acetylcholine (ACh, 1 mM) was added to the serosal solution after 5 min of basal readings and readings continued for a further 5 min. Glucose (10 mM) was then added to the mucosal solution, and readings taken for 10 min. Both mucosal and serosal solutions were then replaced with fresh pre-warmed Krebs buffer. The procedure was repeated for the mucosal solution to remove all traces of glucose. Peptide (NDPK-A or NDPK-B 36–54, 100 μM) was added to both mucosal and serosal solutions and after 30 min stabilization, readings were taken for a further 10 min. Both mucosal and serosal solutions were then replaced with fresh pre-warmed Krebs buffer. Glucose (10 mM) was added to the mucosal solution, and readings taken for 10 min.

### Immunoprecipitation

Membrane proteins were re-suspended in immunoprecipitation buffer (10 mM Tris-HCl pH 7.4, 2 mM EDTA, 1 mM NaF, 1 mM DTT, 1% sodium deoxycholate, 1% NP-40, 0.3 μM aprotonin, 0.2 μM PMSF). The mixture was pre-cleared with protein G-Sepharose beads (30 min at 4°C), centrifuged at 4°C at 350 g for 5 min and the supernatant incubated with primary antibody for 60 min at 4°C. New beads were added and the mixture incubated overnight at 4°C. The incubation mixture was centrifuged at 350 g for 5 min and the pelleted beads washed in 1 ml RIPA buffer (50 mM Tris-HCl pH 7.4, 1% NP-40, 0.5% sodium deoxycholate, 5 mM EDTA, 1M NaCl). This wash step was repeated three times and then 50 μl of 5x Laemmli buffer containing 100 mM DTT was added to the pellet, boiled for 2 min and finally, spun at 420 g for 5 min. An aliquot (20 μl) was then run on 12.5% polyacrylamide gels and blotted onto PVDF membrane.

### Immunoblotting

Proteins (10–100 μg), separated by SDS-PAGE, were transferred to PVDF membrane (Millipore). Pre-stained markers were used to confirm transfer. The blotted membrane was blocked with 5% w/v non-fat dry milk and incubated at room temperature for 60 min. The blot was washed with 1x TBS 0.1% Tween-20 (4 times for 15 min each) and then probed with appropriate primary antibody. The blots were probed with appropriate Horseradish Peroxidase (HRP) conjugated secondary antibody (1:2000) followed by supersignal™ West Pico chemiluminescent detection (Pierce, UK).

### Overlay or far Western assays

Proteins were separated by SDS-PAGE and blotted onto PVDF membranes. The blotted membrane was blocked with 5% w/v non-fat dry milk, following which the cytosol protein (500 μg) in 1x TBS containing 5% w/v non-fat dry milk was then overlaid onto the membrane and incubated at room temperature for 60 min. The blot was washed with 1x TBS 0.1% Tween-20 (4 times for 15 min each) and then probed with antibody to NDPK-B.

### Laser confocal microscopy

HNE were suspended in complete medium 199 and treated with either forskolin (FSK, 10 μm)/3-isobutyl-1-methylxanthine (IBMX, 100 μM) for 30 min, H-89 (1 μM) or PKI (100 nM) for 5 min prior to the addition of FSK (10 μM)/IBMX (100 μM) for 30 min. Control cells were incubated in complete medium 199 alone. The cells were fixed in 4% paraformaldehyde for 30 min at room temperature and quenched with 100 mM glycine for 30 min. Cells were then permeabilised using 1% Triton X-100 in 1x PBS (pH 7.2) for 30 min at room temperature, washed (x3) and blocked with 1% BSA for 60 min at room temperature. Cells were incubated overnight at 4°C with primary antibody (1:100 anti-nm23-H2 mouse monoclonal) in PBS for 60 min, washed (x3) and then incubated with the secondary antibodies [anti-mouse fluorescein isothiocyanate (Sigma, UK) and anti-rabbit/goat rhodamine (Santa Cruz, CA)] at 1:100 dilution for a further 60 min at room temperature. Cells were then washed 5 times with 1x PBS and re-suspended in glycerol (70%). Slides were examined by laser confocal microscopy (LSM-510; Zeiss, Germany).

### Biotinylation of surface membrane proteins

Surface biotinylation of glycosylated CFTR was performed as previously described [50] with some modifications. Briefly, cells grown to confluency were treated with FSK (10 μM)/IBMX (100 μM) ± PKI (100 nM) for 30 min, washed with ice-cold 1x PBS and then biotinylated using 1mg/ml of EZ-Link sulfo-NHS-SS-biotin for 30 min at 4°C. Free biotin was removed by washing twice with ice-cold 1x PBS containing 0.1% BSA and then with ice-cold 1x PBS. Cells were then scraped in ice-cold homogenisation buffer (containing complete protease inhibitor cocktails) and sonicated as previously [47]. The lysate was centrifuged at 300 g for 2 min and the pellet discarded. Pre-washed avidin agarose beads suspended in PBS were added to the supernatant and incubated for 30 min at room temperature with mild shaking. Avidin-bound complexes were pelleted (350 g) for 2 min and washed five times. Biotinylated proteins were eluted in Laemmli buffer, resolved by SDS-PAGE, and immunoblotted with appropriate antibody.

### Whole cell recordings

Standard patch clamp experiments were used to examine whole cell currents in 16HBE14o- cells grown on plastic coverslips (Hamill et al., 1981). Coverslips were placed in a Perspex bath on the stage of an inverted microscope (Olympus IX70) and voltage protocols driven by an IBM-compatible computer, equipped with a Digidata interface (Axon instruments, USA) and the pClamp software, Clampex 8.0 (Axon Instruments, USA). A List EPC-7 amplifier was used to make recordings.

The bath contained Na^+^ Ringer, which has the composition (in mM): 140 NaCl, 2 CaCl_2_, 1 MgCl_2_, 40 mannitol and 10 HEPES (titrated to pH 7.4 with NaOH). The pipette contained (in mM): 135 CsCl (to inhibit K^+^ channels), 2 EGTA, 2 MgCl_2_, 2 Na_2_ATP and 10 HEPES (titrated to pH 7.4 with CsOH). Whole cell currents were saved onto the hard disk of the computer following low-pass filtering at 5 kHz. Cell potential was clamped at a holding potential of –40 mV and then stepped to between +100 and –100 mV, in –20 mV steps. Cell area was calculated from the capacity transients seen in response to a 20 mV potential step, with membrane capacitance assumed to be 1 μF per cm^2^. Slope conductances for each cell were calculated over the appropriate potential ranges; outward (G_out_) +100 to +20 mV and inward (G_in_) -20 to -100 mV using Ohms law. To determine the magnitude of previously identified CFTR and DIDS-sensitive Cl- conductances [51], whole cell current magnitude was initially measured and then 500 μM DIDS was added to the bath (OR Cl- channel magnitude) followed by 10 μM CFTRinh_172_ (CFTR magnitude) [52].

Before obtaining the whole cell configuration, channels were activated by incubation of cells with FSK (10 μM)/IBMX (100 μM) for 30 min. When the effect of the peptides was tested, cells were incubated in the presence of the peptides (100 μM for each) for 30 min before an additional 30 min in the presence of the peptides plus FSK (10 μM)/IBMX (100 μM). For these experiments, a separate control dataset was obtained in the absence of peptide. These control cells were incubated for 30 min in the control solution (no peptides) before the final 30 min incubation in the presence of FSK (10 μM)/IBMX (100 μM).

### Surface Plasmon Resonance (SPR) analysis

The human recombinant nucleoside diphosphate kinase B isoform (NDPK-B) was prepared and provided by Professor Ioan Lascu. CM5 (carboxyl methyl dextran) sensor chips, 1-ethyl-3-(3-dimethylaminopropyl) carbodiimide hydrochloride (EDC), N-hydroxysuccinimide (NHS), ethanolamine hydrochloride and NaOH pH 8.5 were purchased from GE Healthcare. The buffers used for the experiments are: immobilization buffer, 10mM sodium acetate buffer pH 4.0 to 5,5; HBS-EP buffer, 10 mM HEPES, pH 7.4 containing 150 mM NaCl, 3 mM EDTA and 0.005% (v/v) Surfactant P20.

The CM5 measurements were performed using the BIAcoreTM 3000 optical biosensor (GE Healthcare) of the PurIProB platform (Inserm U1078, Brest, 29218, France). In accordance with the pH-scouting wizard, the ligand (NDPK-B), diluted in 10 mM acetate buffers with different pH (4.0, 4.5, 5.0 and 5.5), was injected on the sensor chip for 2 min at a flow rate of 20 μL/min to determine the appropriate immobilization pH conditions. For covalent immobilization of the NDPK-B ligand on the CM5 sensor chip surface, the sensor surface was activated with a freshly mixed solution of 0.4 M EDC and 0.1 M NHS (1:1) for 7 min at a flow rate of 5 μL/min. Purified NDPK-B (5561.5 RU final), was then injected over the activated chip surfaces in 100 μL of 10 mM sodium acetate pH 5.5 (flow rate: 5 μL/min). The residual active groups were inactivated with 1 M ethanolamine-HCl at pH 8.5 for 7 min (flow rate: 5 μL/min).

For binding measurements, ligand—protein interactions were monitored by injecting the NBD1 domain dissolved at various concentrations in the HBS-EP running buffer at the flow rate of 10 μL/min for 2 min. The binding of NBD1 domains was evaluated 20 s into the dissociation phase. In order to continuously monitor the non-specific background binding of samples to the carboxymethyl dextran substrate, a control flow cell was pre-activated and immediately blocked with ethanolamine without exposure to the ligand. Interactions were estimated by subtracting the response in the blank flow cell from the response in the cell with immobilized NDPK-B. Bovine Serum Albumin (BSA, 500 ng) was injected as a negative control under similar conditions. The interaction analyses were carried out in HBS-EP buffer at 25°C. The SPR data analysis was done by applying the BIAevaluation v.4.1.1 software (BIAcore).

### *In silico* analysis

Protein-protein docking analysis was performed using two FFT-based docking software PIPER [53] and Zdock [54]. The *in silico* experiments were performed using NDPK-B crystal structure (PDB code: 1NUE) as the probe and NBD1 crystal structure (PDB code: 1R0X) as the target protein. 1000 complexes were obtained from both docking algorithms and clusterized using the pairwise RMSD (Root Mean Square Deviation < 2Å) into 4 largest clusters. The final complex was chosen according to the energy scoring function.

Molecular dynamics (MD) simulations of the final complex (parameterized with AMBER99) were performed with NAMD 2.8 [55] in order to verify their stability over time; in particular a 100ns of NPT (1atm, 300K) MD simulation were performed after an equilibration phase of 1 ns (positional restraints were applied on carbon atoms to equilibrate the solvent around the protein).

### Antibodies used in this study

CFTR monoclonal antibody was from Labvision/Neomarkers (Runcorn, Cheshire, UK) and CFTR polyclonal antibody (R&D Systems, Minneapolis, USA). The nm23-H1 antibody was from Autogen Bioclear (Calne Wiltshire, UK) and has been described previously (Muimo et al., 1998). The nm23-H2 monoclonal antibody was from Kamiya Biomedical Company (Seattle, Washington, USA) and the nm23-H2 polyclonal antibodies have been described previously (Ma et al., 2002a; Hippe et al., 2003). Anti-NDPK-A does not recognize NDPK-B, nor vice versa, in western blot and immunostaining.

### Solutions

Osmolality of the experimental solutions was checked using a Roebling osmometer and adjusted to 300 ± 1 mOsm.kg^-1^ H_2_O using mannitol or water as appropriate.

### Statistics

Results are presented as mean ± SEM. Effects of experimental interventions were assessed by Student’s t-test and significance was assumed at the 5% level. Unless otherwise indicated all immunoblots are representative of at least three independent experiments.

## Results

### Distribution of NDPK-A and -B in airway epithelial cells

**Fig 1:**
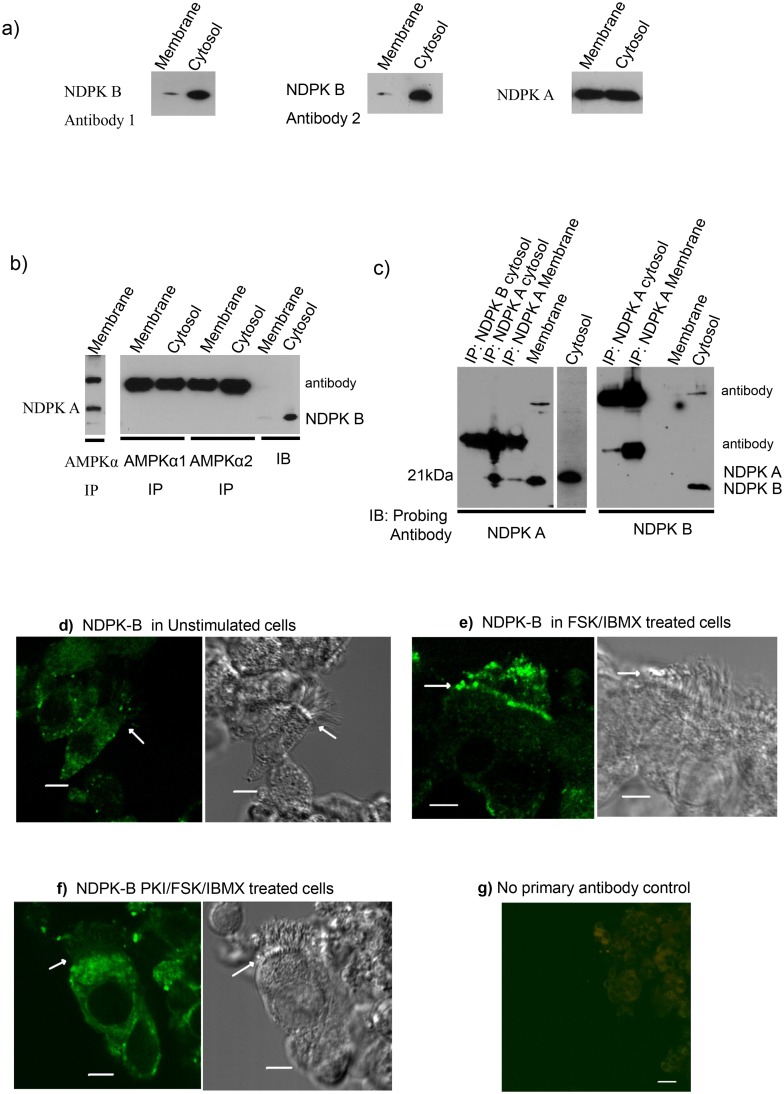
PKA regulates translocation of NDPK-B in airway epithelial cells. **a)** Distribution of NDPK A and NDPK B in 16HBE14o- cells. Western Blot of membrane and cytosol (50 μg) from 16HBE14o- cells separated on a 15% SDS PAGE gel and transferred to PVDF membrane (Left panel, probed with NDPK B rabbit polyclonal antibody (1/5000); Middle panel, probed with NDPK B monoclonal antibody (1/1000); Right panel, probed for NDPK A). **b)** NDPK B is not detected in AMPKα immunoprecipitates. Left panel, western blot of AMPKα (pan) probed for NDPK A. Right panel, immunoprecipitates of AMPKα1 and AMPKα2 probed for NDPK B. **c)** Absence of complex between NDPK A and B in airway epithelia. Left panel, immunoprecipitates of NDPK B and NDPK A probed for NDPK A. Right panel, immunoprecipitates of NDPK A and NDPK B probed for NDPK B. Antibody staining detected with HRP antibody and a chemiluminescent substrate. Results representative of at least four independent experiments. Modulation of PKA activity alters localisation and distribution of NDPK-B in HNE. Immunocytochemical staining of HNE for NDPK-B in cells: **d)** untreated, **e)** treated with FSK/IBMX for 30 min, **f)** treated with PKI (5 min) prior to FSK/IBMX for 30 min, **g)** No primary antibody control. Bar is 5 μM and arrows show position of apical membrane. The result is representative of three independent experiments.

Among NDPK isoforms, NDPK-A and B share the highest sequence homology (88%), and both have been detected in the cytoplasm and in the nucleus in various cell types [28, 56, 57]. However, the properties and function of membrane bound NDPK from mammalian cells is less well characterized. We have previously shown that NDPK-A exists in apical membrane of human and sheep airway epithelia [38] and that it forms a complex with AMPKα1 and CFTR [35]. Western blot analysis shows that NDPK-B is also present in both membrane and cytosol of 16HBE14o- cells and sheep tracheal epithelia. Interestingly, although NDPK-B is detectable in the membrane fraction by immunoblot analysis, it is predominantly cytosolic in 16HBE14o- cells (Fig 1a), sheep tracheal epithelia and rat thalamus (not shown). In contrast, NDPK-A appears to be more uniformly distributed between the membrane and cytosol fractions of 16HBE14o- cells (Fig 1a), sheep tracheal epithelia and rat thalamus (not shown). Previous studies have demonstrated that NDPK-A and -B form heterohexamers in human erythrocytes [28]. However, since our data shows that the membrane fraction in airway epithelia contains disproportionate amounts of NDPK-A and, NDPK-B the two isoforms may not always exist as hetero-tetramers/hexamers or share the same subcellular distribution in airway epithelia.

### Do NDPK A & NDPK B form complexes in airway epithelia?

The differential subcellular distribution of NDPK A and NDPK B in 16HBE14o- cells suggests that the two isoforms are probably involved in different cellular processes. We have recently provided evidence that NDPK A forms a functional complex with AMPKα [40]. Analysis of AMPKα1 or AMPKα2 immunoprecipitates from membrane and cytosol of 16HBE14o- cells or sheep tracheal epithelium (not shown) for the presence of NDPK-B (Fig 1b), shows that while NDPK A co-immunoprecipitates with AMPKα1 (left panel), NDPK-B is undetectable. Equally, AMPKα is undetectable on western blots containing immunoprecipitates of NDPK-B (not shown). This suggests that NDPK B exists independently of the NDPK A/AMPKα1 complex. Furthermore, we investigated complex formation between NDPK A and NDPK B in both membrane and cytosol of airway epithelia. Western blot analysis of NDPK-A and NDPK B immunoprecipitates from airway epithelia shows that NDPKA is undetectable in NDPK B immunoprecipitates and vice versa (Fig 1c). The failure to co-immunoprecipitate NDPKA and NDPK B in airway epithelia indicates that the two proteins may not exist as heterohexamers under normal cellular conditions in these cells. Furthermore, this data also suggests that the two proteins are likely to localise to different subcellular compartments and engage in different cellular events in airway epithelia.

### NDPK-B interaction with CFTR is regulated by cAMP/PKA

Previous work shows that NDPK binds cAMP *in vitro* [44, 58]. To analyse whether cAMP might affect cellular distribution and function of NDPK-B in airway epithelial cells, we immunolocalised NDPK-B in human nasal brushings treated with or without the well-established stimulus that raises cyclic AMP (a combination of the adenylyl cyclase stimulator forskolin (FSK) and a phosphodiesterase inhibitor (IBMX) to prevent cAMP degradation). Fig 1d shows confocal microscopy analysis of unstimulated human nasal epithelial cells stained with a selective anti-NDPK-B antibody. Although NDPK-B staining was observed throughout the cell including the nucleus, it was predominantly located within the cytoplasm within distinct punctuate structures (as noted by others (18)). Following cell stimulation with FSK (10 μM)/IBMX (100 μM), NDPK-B staining predominates at the apical membrane (Fig 1e) and is unexpectedly also found associated with cilia suggestive of a secretory event (this was not explored further). To determine whether PKA was involved, we inhibited PKA activity using PKI, a cell permeable and selective peptide pseudo-substrate inhibitor of the catalytic activity of PKA (100 nM myristoylated protein kinase A inhibitor amide 14–22 − (54)), added prior to FSK/IBMX stimulation. PKA inhibition maintained the pre-stimulated pattern of NDPK-B staining within the cytoplasm (Fig 1f). This suggests that PKA might regulate the distribution and or translocation/secretion of NDPK-B in airway epithelia.

**Fig 2:**
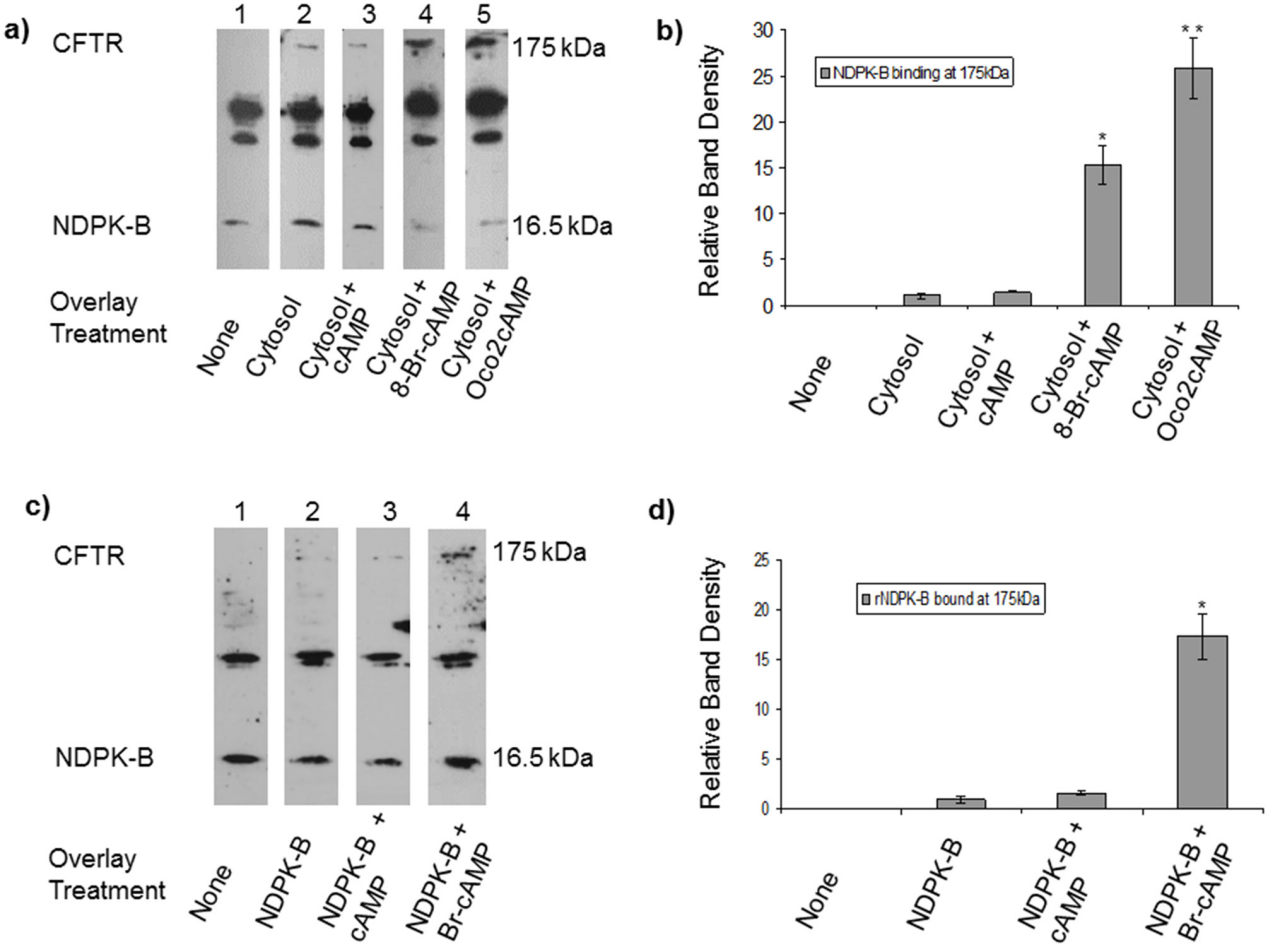
NDPK-B interaction with CFTR is regulated by cAMP. **a**) cAMP-dependent NDPK-B binding to a 175-kDa protein in overlay assays. Identical immunoblots of (100 μg) probed for NDPK-B. Lanes: 1) control blot with no overlay, 2) blot overlaid with solution containing 16HBE14o- cytosol proteins (0.5 mg). 3, 4, 5) Blot overlaid with solution 2 above containing cAMP (100 μM), 8-Br-cAMP (100 μM) or N6,O2'- dioctanoyl-cAMP (Oco2cAMP) (100 μM), respectively. Cyclic AMP enhanced NDPK-B staining at 175 kDa. Results are representative of four separate experiments. **b)** Quantification of the band density of NDPK-B/CFTR complex at 175kDa (n = 4) * P<0.001 Student *t*-test. Dioctanoyl cAMP increased NDPK-B binding 20-fold. **c)** Recombinant NDPK-B binds to a 175-kDa protein in cAMP-dependent manner in overlay assays. Identical western blots of membrane proteins from 16HBE14o- cells (100 μg) probed for NDPK-B staining. Lanes 1) Control with no overlay. 2) Blot overlaid with solution containing 1 μg of recombinant NDPK-B. 3) Overlaid with solution 2 containing cAMP (100 μM), 4) overlaid with solution 2 containing 8-Br-cAMP (100 μM). **d**) Quantification of the band density of NDPK-B at 175kDa (n = 4) * P<0.001 Student *t*-test. Br-cAMP increased NDPK-B binding 20-fold.

**Fig 3:**
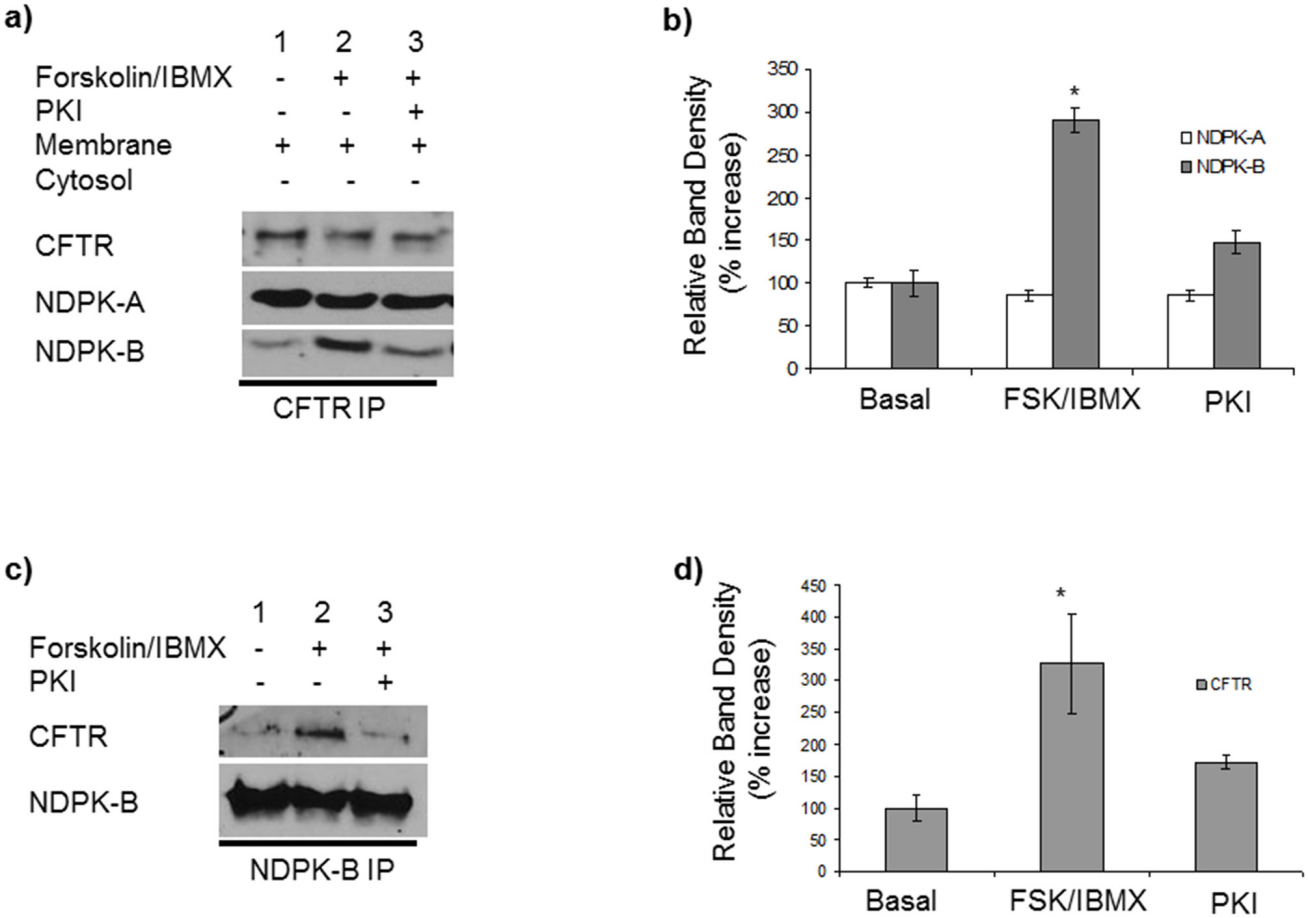
Forskolin (FSK) enhances NDPK-B interaction with CFTR in 16HBE14o- cells **a)** PKA regulates co-immunoprecipitation of CFTR with NDPK-B in 16HBE14o-. Immunoblot of CFTR immunoprecipitate from 16HBE14o- membranes showing that FSK increases the amount of NDPK-B, but not NDPK-A, which co-immunoprecipitates with CFTR. Cells were untreated (lane 1), treated with FSK/IBMX for 30 min (lane 2) or with PKI for 5 min prior to FSK/IBMX treatment (lanes 3) and probed for NDPK-A and NDPK-B. Equal loading of the CFTR immunoprecipitate, was confirmed by re-probing the same blot for CFTR using a polyclonal antibody (R & D systems). **b)** Quantification of the band density of NDPK-B and NDPK-A shows a 3-fold increase in NDPK-B co-immunoprecipitation with CFTR with FSK/IBMX (n = 4). * P<0.05 Student *t*-test. **c)** Immunoblot of NDPK-B immunoprecipitate from 16HBE14o- membrane from cells: untreated (lane 1); treated with FSK/IBMX (lane 2) or PKI (5 min) prior to FSK/IBMX for a further 30 min (lane 3) and probed for CFTR. Equal loading of immunoprecipitate was confirmed by re-probing the same blot for NDPK-B polyclonal antibody. PKI inhibited the impact of FSK/IBMX on NDPK-B co-immunoprecipitation with CFTR. **d)** Quantitative analysis of the CFTR band density shows a 2-fold increase in CFTR co-immunoprecipitation with NDPK-B with FSK/IBMX (n = 4) * P<0.05 Student *t*-test.

CFTR binding to cAMP has been proposed as a regulator of channel activation [59]. Since cAMP and PKA are cooperative regulators of CFTR (4, 5) and mature CFTR is apically localised in airway epithelia [60], we analysed the interaction between CFTR and NDPK-B using cell lysates. Fig 2a shows additional NDPK-B staining occurs at 175 kDa, when Western blots of SDS-PAGE-separated membrane proteins from (CFTR-expressing) immortalised 16HBE14o- cells (100 μg) or ovine tracheal epithelial membranes (not shown) are overlaid with a solution containing cytosol from the relevant cognate cells (Far-Western blot). Addition of non-hydrolysable analogues of cAMP (8-bromo or di-octanoyl cAMP, 100 μM) to the overlay solution strikingly increased NDPK-B staining at 175 kDa nearly 20-fold (Fig 2a and 2b). This result strongly suggested cAMP might control the assembly of an NDPK-B/CFTR protein complex at the apical membrane of airway epithelia. In order to determine whether the interaction between NDPK-B was direct or involved a bridging protein, a Western blot of a CFTR immunoprecipitate was first overlaid with purified NDPK-B and then probed for NDPK-B. Enhanced NDPK-B staining is observed at 175 kDa in the presence of cAMP (100 μM) (Fig 2c and 2d). To test the selectivity of the cAMP effect, we studied NDPK-A, and found that although NDPK-A also binds at the expected molecular weight for CFTR in overlay assays, the binding of this highly homologous family member is not enhanced by cAMP (not shown, see also Fig 3a and 3b).

Western blot analysis of CFTR immunoprecipitates from membranes of 16HBE14o- cells stimulated with FSK/IBMX shows that FSK significantly enhanced the amount of NDPK-B co-immunoprecipitating with CFTR (Fig 3a, lane 2; quantitation Fig 3b). Pre-treatment of the cells with PKA inhibitors, PKI (100 nM) or H-89 (1 μM, not shown) for 5 min prior to stimulation with FSK/IBMX for 30 min, reduced the amount of NDPK-B interacting with CFTR (Fig 3a and 3b). Similarly, FSK-stimulation significantly increases the amount of CFTR co-immunoprecipitating with NDPK-B (Fig 3c and 3d). On the other hand, analysis of NDPK-A staining in CFTR immunoprecipitates shows no variation following cell stimulation with either FSK/IBMX or PKI/FSK/IBMX suggesting that unlike NDPK-B, NDPK-A interaction with CFTR is not regulated by changes in cellular cAMP/PKA activity (Fig 3a and 3b). Thus, our data suggests cAMP and PKA selectively mediate promote the NDPK-B interaction with CFTR and that despite its similar sequence, NDPK-A is not part of this process.

### NDPK-B associates only with Cell surface CFTR

**Fig 4:**
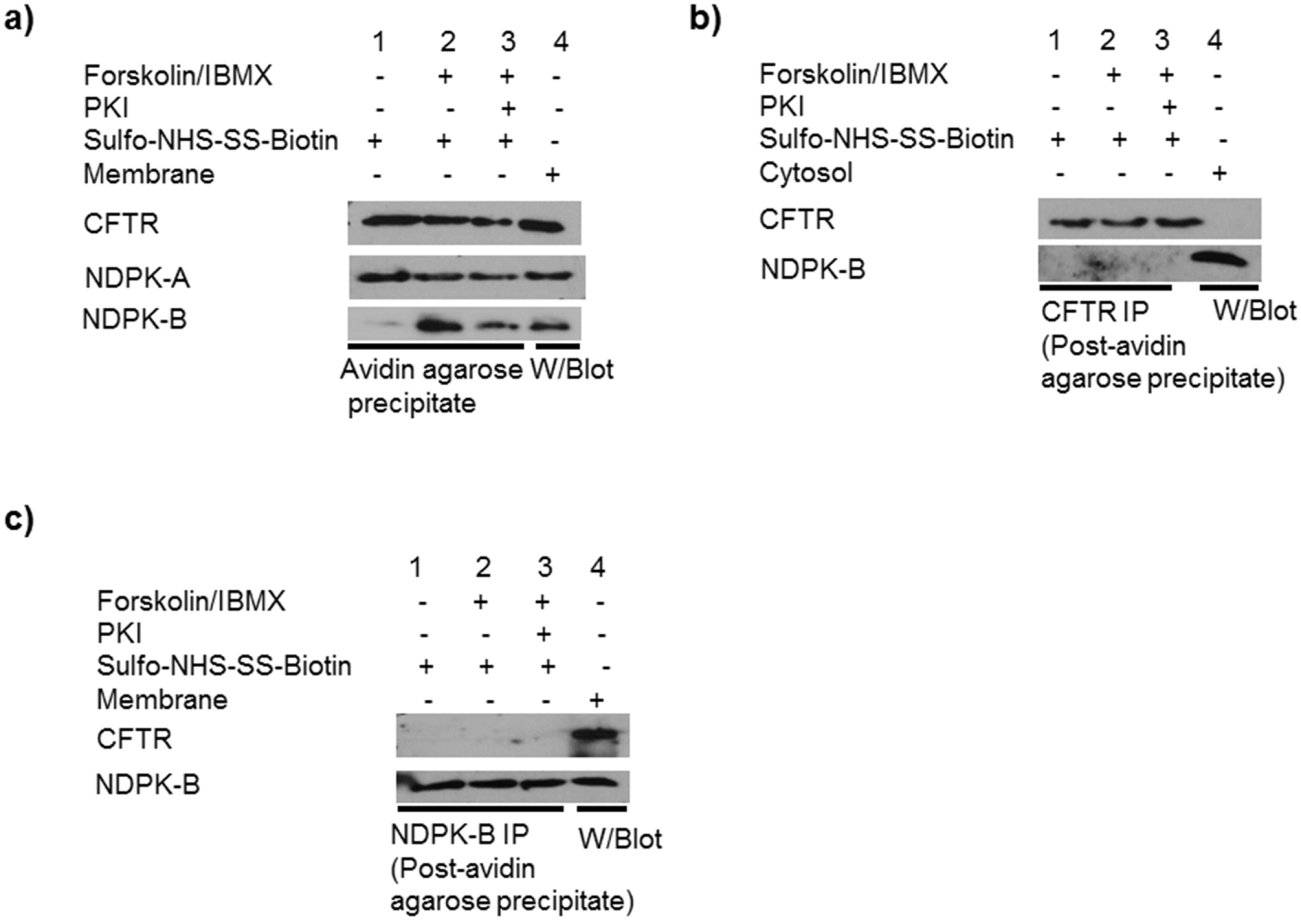
NDPK-B binds cell surface CFTR in forskolin/IBMX stimulated cells. **a**) Immunoblots of avidin-agarose precipitates from lysates of 16HBE14o- cells ± FSK or PKI/ FSK/IBMX, biotinylated for 30 min at 4°C and probed for CFTR, NDPK-A and NDPK-B. **b**) Non-cell surface CFTR does not associate with NDPK-B. Immunoblots of CFTR immunoprecipitates from lysates of 16HBE14o- cells ± FSK or PKI/FSK/IBMX (post the avidin precipitation described in A, above) probed for CFTR and NDPK-B. **c**) NDPK-B does not associate with non-cell surface CFTR. Immunoblots of NDPK-B immunoprecipitates from lysates of 16HBE14o- cells ± FSK or PKI/FSK/IBMX (post the avidin-agarose precipitation described in A, above) probed for CFTR and NDPK-B. To confirm equal loading of the immunoprecipitates, blots were stripped and re-probed with CFTR rabbit polyclonal antibody (R & D systems) (B) or NDPK polyclonal antibody (C). The results are representative of two independent experiments.

The cAMP/PKA-dependent translocation of NDPK-B to the apical membrane suggested that NDPK-B might associate with cell surface CFTR. In order to confirm that the cAMP/PKA-induced complex is associated with the cell surface CFTR and not CFTR within intracellular organelles, 16HBE14o- cells were surface biotinylated with cell-impermeant EZ-Link sulfo-NHS-SS-biotin at 4°C for 30 min. Fig 4a (lane 2) shows increased levels of NDPK-B co-precipitate with avidin-agarose in cells treated with FSK/IBMX. On the other hand, inhibition of cellular PKA activity with PKI, prior to FSK/IBMX stimulation, reduces the amount of NDPK-B precipitating with avidin-agarose (Fig 4a lane 3). This data is consistent with the notion that NDPK-B translocation to the apical membrane leading to an association with cell surface CFTR. The selectivity of this binding is demonstrated by our observing no change in the levels of NDPK-A or CFTR co-precipitating with avidin-agarose following cell treatment with FSK/IBMX (Fig 4a). To assess whether NDPK-B also associates with non-cell surface CFTR, CFTR immunoprecipitates from the residual cell extracts (± FSK/IBMX-stimulation) already depleted of cell surface/integral membrane proteins by avidin precipitation (post-avidin supernatant) were probed for NDPK-B. Fig 4b and 4c show that, despite the presence of both proteins in the post-avidin supernatant, NDPK-B did not co-immunoprecipitate with CFTR from this fraction. Similarly, in the reverse experiment, CFTR staining is undetectable in NDPK-B immunoprecipitates from supernatant depleted of biotin-labelled proteins. Thus, following cAMP/PKA stimulation, there was increased association of NDPK-B, but not NDPK-A, with cell surface CFTR. Complex formation is not observed with non-cell surface CFTR suggesting that NDPK-B tethers to cell surface CFTR.

### NDPK-B interaction domain

**Fig 5:**
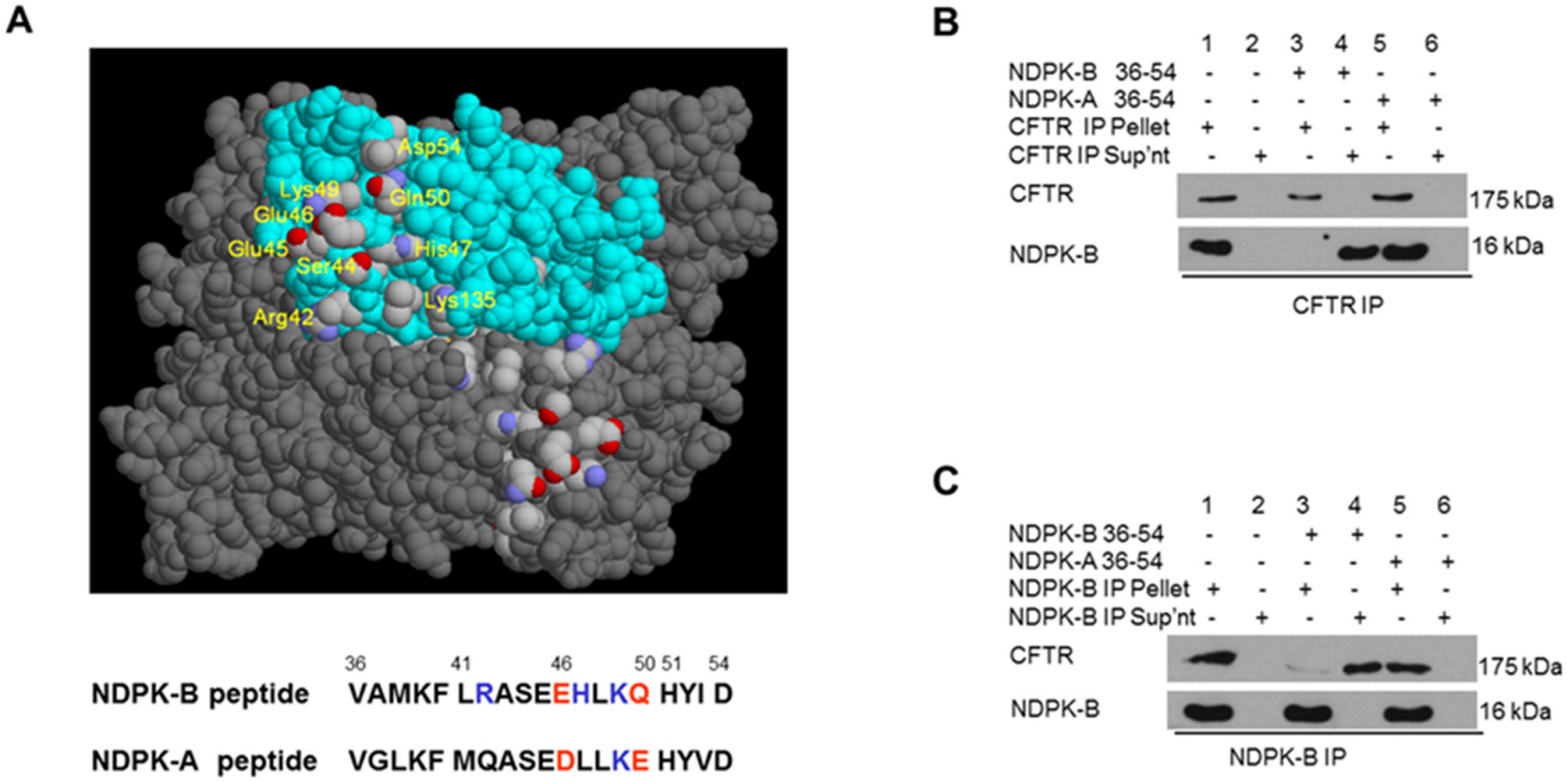
Analysis of NDPK-B interaction with CFTR. **a)** Position of the exposed side-chains of peptide 36–54, based on the published crystal structure of the NDPK-B [81]. The CPK code was used: nitrogen is blue and oxygen is red. One subunit is shown in cyan to identify its border. Figure generated with RASMOL. **b)** Immunoblots of CFTR immunoprecipitates from lysates of 16HBE14o- cells treated with FSK/IBMX, and probed for NDPK-B and CFTR show peptide NDPK-B 36–54 (lanes 3, 4), but not peptide NDPK-A 36–54 (lanes 5, 6), released NDPK-B from CFTR immunoprecipitate. Control incubations with buffer alone are shown in lanes 1, 2. **c)** Peptide NDPK-B 36–54 releases CFTR from complex with NDPK-B. Immunoblots of NDPK-B immunoprecipitates from lysates of 16HBE14o- cells treated with FSK/IBMX, and probed for CFTR and NDPK-B show peptide NDPK-B 36–54 (lanes 3, 4), but not buffer alone control (lanes 1, 2) or peptide NDPK-A 36–54 (lanes 5, 6), released CFTR from NDPK-B immunoprecipitate.

NDPK-A and NDPK-B are reported to exist as heterohexamers and yet we find a difference in their localization. In order to identify the NDPK-B domain responsible for interaction with CFTR, we generated a 19-mer peptide corresponding to one of the regions of NDPK-B showing the least sequence homology to NDPK-A comprising amino acids 36–54 (Fig 5a). Immunoblot analysis shows that NDPK-B is released into the supernatant of the beads bearing the CFTR immunoprecipitate and exposed to the peptide (Fig 5b lanes 3, 4, compare with lane 1). These immunoprecipitates were from lysates of 16HBE14o- cells stimulated with FSK/IBMX and incubated with the peptide NDPK-B 36–54 (V_36_AMKFLRAS_44_EEHLKQHYID_54_) for 30 min at 30°C. In control incubations, without peptide (Fig 5b lanes 1, 2) or with the corresponding peptide from NDPK-A 36–54 (V_36_GLKFMQASEDLLKEHYVD_54_) (Fig 5b lanes 5, 6), release of NDPK-B from the CFTR immunoprecipitate was never observed. The selective release of NDPK-B induced by peptide NDPK 36–54 indicates that this peptide disrupted the NDPK-B/CFTR complex and suggests this region of NDPK-B, may constitute a key NDPK-B interaction domain for CFTR. Similar results were obtained in the reverse experiment, the peptide NDPK 36–54 -induced CFTR release from beads containing the NDPK-B immunoprecipitate (Fig 5c).

### CFTR binding site for NDPK-B

**Fig 6:**
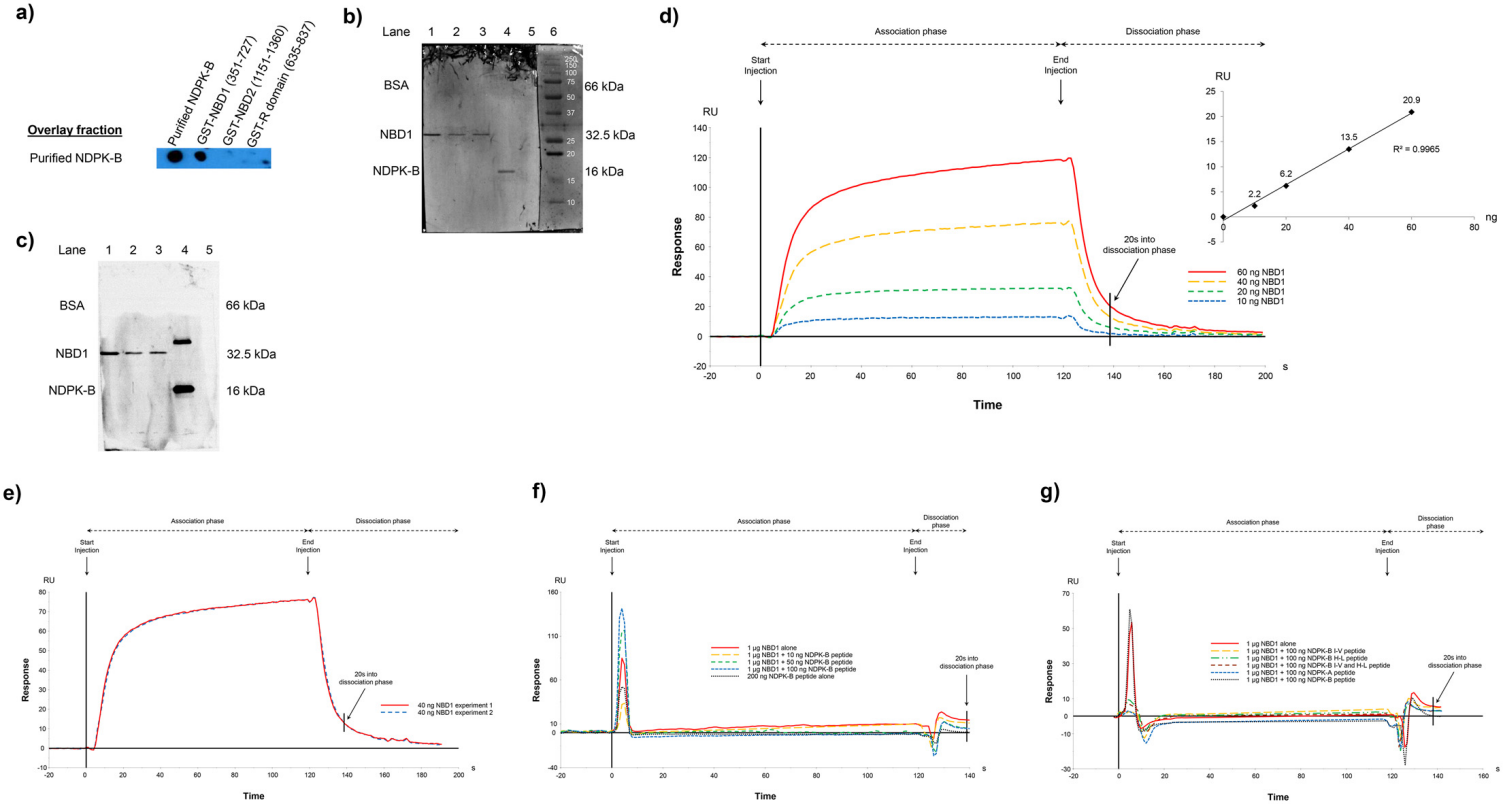
NDPK-B /NBD1 interaction analysis of by Surface Plasmon Resonance. **a**) Membrane (PVDF) was spotted with purified NDPK-B (lane 1), GST-NBD1 (351–727), R domain (635–837) and GST-NBD2 (1151–1360) was overlaid with purified recombinant NDPK-B and probed for NDPK-B. Purified NDPK-B bound to GST-NBD1. **b**) Coomassie blue staining of PVDF membrane showing the purity of NBD1 (28 kDa) (lanes 1–3; 900, 300 and 100 ng per lane, respectively) and NDPK-B (lane 4, 16 kDa) and the amounts of the proteins loaded. **c**) Overlay analysis of the direct interaction between the NBD1 domain (351–727) of CFTR and NDPK-B. Overlay experiment showing western blot containing NBD1 (lanes 1–3; 900, 300 and 100 ng per lane, respectively), NDPK-B (250 ng, lane 4) and BSA (250 ng, lane 5) overlaid with purified NDPK-B (50 μg) and then probed for NDPK-B staining using NDPK-B specific antibodies. BSA was used as a control to exclude non-specific interactions with NDPK-B. **d)** SPR analysis showing the direct interaction between the NBD1 of CFTR and NDPK-B. Example of sensorgrams obtained when several quantities of His-tagged NBD1 (10, 20, 40 or 60 ng) were injected over immobilized NDPK-B proteins. In the inset, the RU = f(ng) curve shows that the RU values obtained twenty seconds into the dissociation phase linearly increase with the amount of injected NBD1. **e)** SPR analysis showing the repeatability of the measurement of the direct interaction between the NBD1 of CFTR and NDPK-B. Example of sensorgrams obtained from two separate measurements when 40 ng of His-tagged NBD1 were injected over immobilized NDPK-B proteins. **f)** SPR analysis showing the specificity of the interaction between the NBD1 of CFTR and NDPK-B. Example of sensorgrams obtained when 1 μg of untagged NBD1 was injected alone or with several quantities of NDPK-B peptide (10, 50 or 100 ng) over immobilized NDPK-B proteins. A sensorgram obtained when 200 ng of NDPK-B peptide was injected alone over immobilized NDPK-B proteins is also shown. **g)** SPR analysis showing the NDPK-B key residues for the NBD1-NDPK-B interaction. Example of sensorgrams obtained when 1 μg of untagged NBD1 was injected alone or with 100 ng of different mutated NDPK-B peptides over immobilized NDPK-B proteins. Sensorgrams obtained when 1 μg of untagged NBD1 was injected with 100 ng of NDPK-A or NDPK-B peptide over immobilized NDPK-B proteins are also shown.

In order to establish whether NDPK-B interacts directly with CFTR, we generated fusion proteins of various CFTR domains: nucleotide binding domain 1 (NBD1, aa 351–727), R-domain (635–837) and nucleotide binding domain 2 (NBD2, 1151–1360) and localised NDPK-B binding to NBD1 by overlay on dot- blots (Fig 6a). Fig 6b shows the purity (by Coomassie blue staining) of untagged NBD1 (various amounts) and of purified NDPK-B protein used for overlay analysis. Fig 6c: Western blot and overlay analysis indicating direct interaction between pure untagged NBD1 and NDPK-B (PVDF membrane containing untagged NBD1 was overlaid with purified NDPK-B and then probed with anti-NDPK-B). The interaction was then confirmed by Surface Plasmon Resonance (SPR) (Fig 6d–6g) assays. We injected several quantities of His-tagged NBD1 (10, 20, 40 or 60 ng) over immobilized NDPK-B proteins and showed the direct interaction between the NBD1 of CFTR and NDPK-B (Fig 6d–6g). The RU values obtained twenty seconds into the dissociation phase were 2.2, 6.2, 13.5 and 20.9 for 10, 20, 40 and 60 ng of NBD1 respectively, and as such, increased linearly with the amount of injected NBD1 (Fig 6d). We evaluated the repeatability of the measurement of the direct interaction between the NBD1 of CFTR and NDPK-B in two separate experiments whereby 40 ng of His-tagged NBD1 injected over immobilized NDPK-B proteins provided a similar number of RU in both cases (13.4 and 13.6) (Fig 6e). This showed the repeatability of the measurement.

To assess the specificity of the NBD1-NDPK-B interaction we used a 19 amino acid (36–54: VAMKFLRASEEHLKQHYID) synthetic peptide of NDPK-B (Fig 5a) that disrupts the NDPK-B/CFTR complex (Fig 5b and 5c) under similar conditions. We injected 1 μg of untagged NBD1 alone or with several quantities of NDPK-B peptide (10, 50 or 100 ng) over immobilized NDPK-B proteins and observed that the number of RU decreased with the amount of injected NDPK-B peptide showing the specificity of the interaction between the NBD1 of CFTR and NDPK-B (Fig 6f). As a control we also injected 200 ng of NDPK-B peptide alone over immobilized NDPK-B proteins and observed no interaction confirming the specificity of the interaction between the NBD1 of CFTR and NDPK-B (Fig 6f). First, our SPR data show a specific interaction between the NBD1 of CFTR and NDPK-B, confirming the overlay analysis (Fig 5a–5c). Secondly, these SPR results confirm the ability of NDPK-B peptide to disrupt the NDPK-B/CFTR complex, showing that this region of NDPK-B (amino acid 36–63) may constitute the NDPK-B interaction domain for CFTR.

To identify the NDPK-B key residues for the NBD1-NDPK-B interaction, we used different mutated NDPK-B peptides as follows: NDPK-B peptide with I-V substitution, NDPK-B peptide with H-L substitution and NDPK-B peptide with both I-V and H-L substitutions. By looking at the sequence, we Figured residues I or H or both might be key to the interaction and that the substitution may affect the ability of NDPK-B peptide to disrupt interaction and cause it to behave like NDPK-A peptide which doesn’t disrupt the NDPK-B interaction with CFTR (Fig 5b and 5c). We injected 1 μg of untagged NBD1 alone or with 100 ng of different mutated NDPK-B peptides over immobilized NDPK-B proteins and observed a similar signal in all cases showing that theses amino acids substitutions affect the ability of NDPK-B peptide to disrupt the NBD1-NDPK-B interaction (Fig 6g). We also injected 1 μg of untagged NBD1 with 100 ng of NDPK-A peptide over immobilized NDPK-B proteins and obtained an equivalent response showing that these residues substitutions cause the mutated NDPK-B peptides to behave like NDPK-A peptide (Fig 6g). These data suggest that I and H amino acids are key residues for the NBD1-NDPK-B interaction. As a control we finally injected 1 μg of untagged NBD1 with 100 ng of NDPK-B peptide over immobilized NDPK-B proteins and observed a dissimilar signal, with a number of RU that became insignificant confirming the ability of NDPK-B peptide to disrupt the NDPK-B interaction with CFTR (Fig 6g). These SPR results first confirm the ability of the NDPK-B peptide to disrupt the CFTR/NDPK-B complex and secondly identify that I and H amino acids as key residues for this NBD1-NDPK-B interaction (NB. The transient signals are artefacts caused by the modification of the refractive index due to buffer change just after the injection start/stop. These artefacts do not alter the analysis (RU values are obtained twenty seconds into the dissociation phase)).

**Fig 7:**
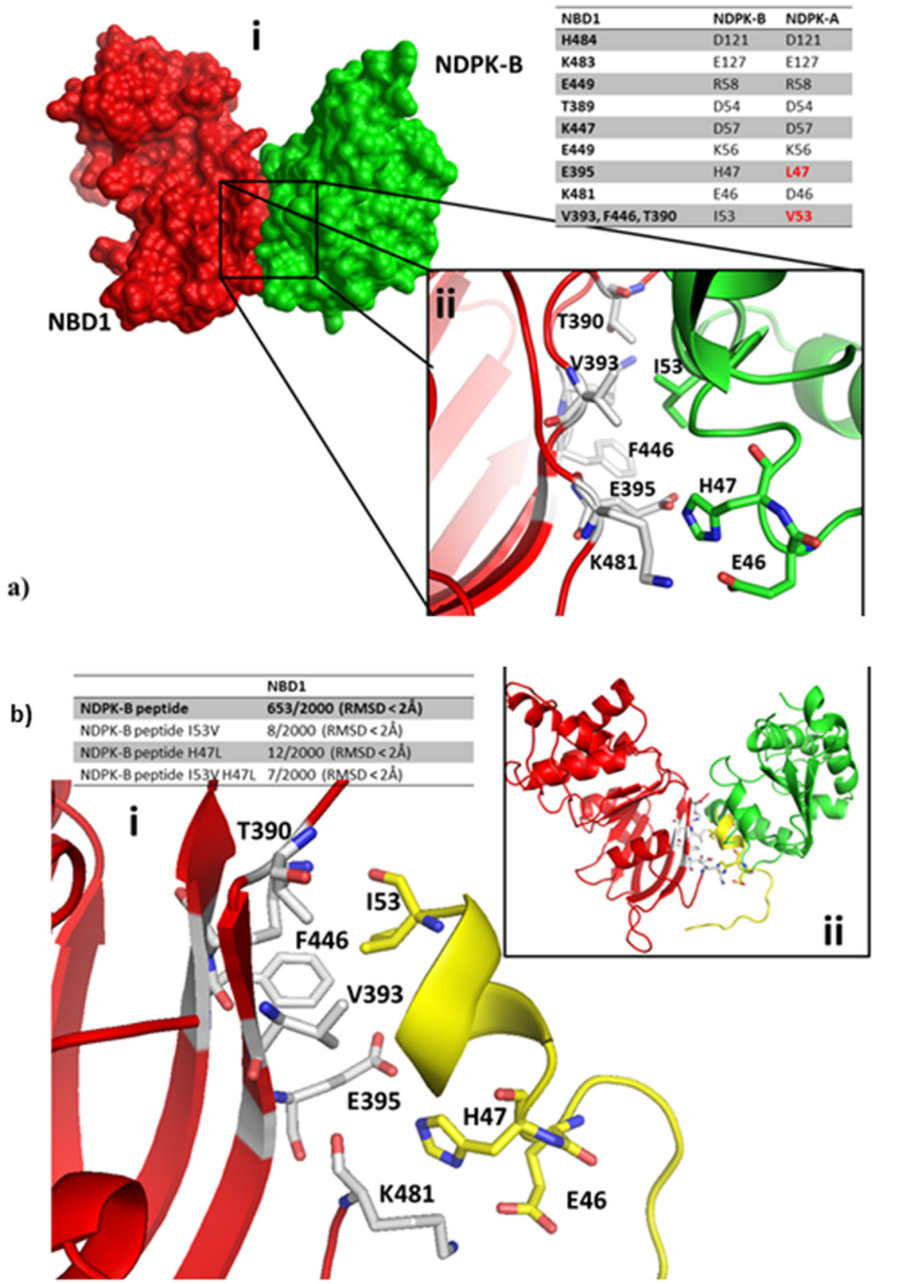
NDPK-B and NBD1 protein-protein docking analysis. **a**) **i)** Protein-protein docking complex between NBD1 (red) and NDPK-B (green); Analytic Connolly’s surface has been highlighted. **ii)** Detailed interaction pattern between NBD1 and NDPK-B. Table inset: differences between NDPK-B and NDPK-A at the interaction surface. **b)**. **i)** Detailed interaction pattern between NBD1 (red) and the peptide 36–51 from NDPK-B (Yellow). **ii)** Superposition between 36–51 peptide (yellow) and NDPK-B full protein (green). Table inset: statistical analysis of NBD1-NDPK peptides complexes.

To clarify the binding motif of NBD1 and NDPK-B, protein protein docking experiments were performed by using two FFT based software, PIPER [53] and Zdock [54] (see Methods section). As shown in Fig 7a, the complex between NBD1 and NDPK-B involves a deep interaction surface area (693 Å^2^). Electrostatic interactions are predominant, however a small hydrophobic pattern has been revealed by *in silico* analysis. In detail, NDPK-B I53 is submerged into a small hydrophobic pocket formed by V393, F446 and the methyl group of T390 of NBD1. This hydrophobic zone is surrounded by different electrostatic interactions, namely between NBD1 K447 and NDPK-B D57, and between NBD1 E395 and NDPK-B H47, furthermore interacting also with NDPK-B E46. On the other hand, no significant results was obtained from the protein-protein docking analysis between NBD1 and NDPK-A, under the same experimental condition used in the case of NDPK-B (see Methods section). Interestingly NDPK-A presents a couple of substitutions in two key points of the interaction pattern revealed by the *in silico* analysis of NBD1 and NDPK-B: the NDPK-B I53 and H47 are substituted with V53 and L47 in the case of NDPK-A as shown in the table inset of Fig 7a.

Significant results were obtained also by studying the interaction between NBD1 and the peptide 36–54 (VAMKFLRASEEHLKQHYID) derived from NDPK-B. The complex obtained from the protein-protein docking analysis is partially superimposable with the complex between NBD1 and the full NDPK-B protein (Fig 7b). The *in silico* results suggest that the peptide 36–54 could directly and specifically compete with the interaction between NBD1 and NDPK-B, by sharing the same binding zone, thus disrupting NDPK-B/CFTR complex. On the other hand, the protein-protein docking analysis between NBD1 and the NDPK-B peptide carrying the single or the double I-V/H-L substitutions does not reveal statistically significant complexes formation, thus confirming that this peptide, characterized by the NDPK-A substitutions, is not able to interact as efficiently as the NDPK-B WT peptide. In particular, as shown in the table inset of Fig 7b, while the NBD1-NDPK-B WT peptide complex present a high statistical significance (653 complexes with an RMSD under 5Å), all the substituted peptides are not able to produce statistically relevant complexes.

### NDPK-B and CFTR function

**Fig 8:**
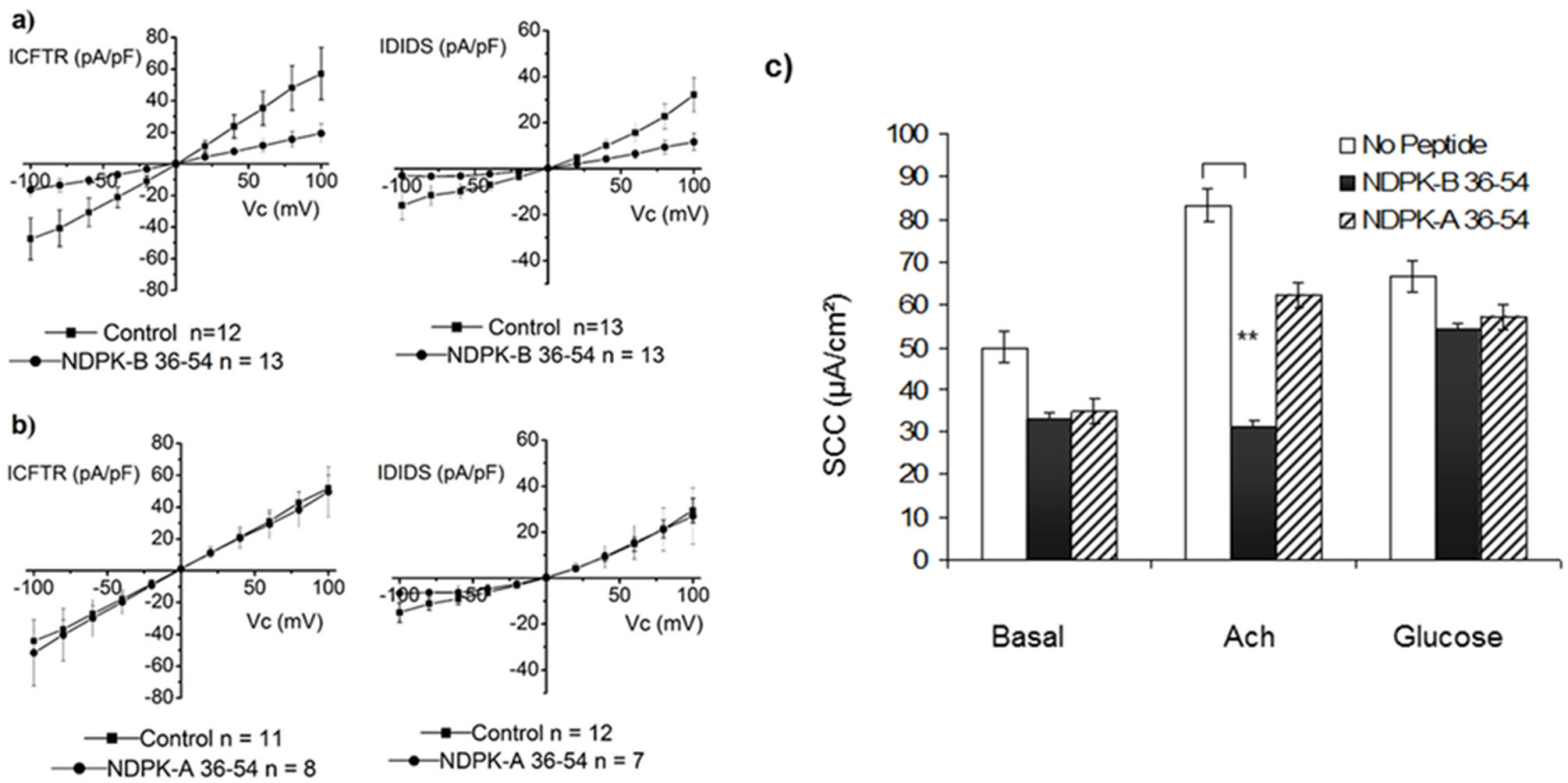
Effect of disruption of the NDPK-B/CFTR complex on the magnitude of FSK-dependent IDIDS and ICFTR in 16HBE14o- cells and the ACh-induced SCC in intestinal biopsy. **a)** Effect of peptide NDPK-B 36–54 on ICFTR and IDIDS. Cells were incubated with NDPK-B 36–54 (100 μM) for 30 min prior to exposure to FSK/IBMX plus peptide NDPK-B 36–54 for 30 min. Control currents for each dataset were time and day matched. **b)** Lack of effect of peptide NDPK-A 36–54 on the outward DIDS-sensitive and CFTR_ihn172-_ sensitive conductances. Cells were incubated for 30 min in the presence of the peptide (100 μM), followed by incubation with the peptide plus FSK and IBMX for 30 min. Control currents for each dataset were time and day matched. **c**) Effect of peptide NDPK-A or NDPK-B 36–54 on SCC in gut epithelia. SCC measurements obtained in response to ACh (Cl^-^ flux) and glucose (Na^+^ flux) stimulation of mounted gut epithelia biopsies. Measurements were taken in the presence or absence of the synthetic peptides NDPK-A or NDPK-B 36–54 (100 μM) (n = 3) **P<0.05 ANOVA.

Previous studies have demonstrated that NDPK regulates muscarinic K^+^ and calcium -activated K^+^ (KCa3.1) channels [33, 41]. To determine the functional significance of the cAMP/PKA-dependent cell surface associated NDPK-B/CFTR complex, we analysed the impact of peptide NDPK-A 36–54 or peptide NDPK-B 36–54 on whole cell currents in 16HBE14o- cells. Fig 8a, shows that peptide NDPK-B 36–54, which disrupts the NDPK-B/CFTR complex (see Fig 4), reduces the magnitude of both the DIDS-sensitive and CFTR-mediated chloride conductances. The CFTR and OR mediated conductances were significantly smaller in the presence of the peptide. The CFTR-sensitive G_out_ was 574 ± 164 μS/cm^2^ (n = 12) versus 187 ± 57.6 μS/cm^2^ (n = 13), in the absence and presence of NDPK-B 36–54, respectively. The CFTRinh-sensitive G_in_ was 465 ± 132 μS/cm^2^ (n = 12) versus 165 ± 49.4 μS/cm^2^ (n = 13). The DIDS-sensitive G_out_ was 341 ± 77.6 μS/cm^2^ (n = 13) versus 119 ± 37.9 μS/cm^2^ (n = 13), in the absence and presence of NDPK-B 36–54, respectively. The DIDS-sensitive G_in_ was 172 ± 66.3 μS/cm^2^ (n = 13) versus 23.9 ± 13.8 μS/cm^2^ (n = 13). This suggests that the NDPK-B/CFTR complex is functionally significant and that NDPK-B regulates both the cAMP/PKA-dependent CFTR and the ORCC-mediated currents in epithelia. Since NDPK-A forms a complex with AMPKα independently of NDPK-B in airway epithelia (25), we also analysed the impact of peptide NDPK-A 36–54 on CFTR function. As expected, peptide NDPK-A 36–54, which does not disrupt the NDPK-B/CFTR complex, was without effect on both the cAMP/PKA-dependent ORCC and CFTR-mediated currents (Fig 8b). The CFTR and OR mediated conductances were not significantly different in the presence of the peptide. (n = 11 or 12 as stated). The CFTRinh-sensitive G_out_ was 510 ± 85.0 μS/cm^2^ (n = 11) versus 486 ± 149 μS/cm^2^ (n = 8), in the absence and presence of NDPK-B 36–54, respectively. The CFTRinh-sensitive G_in_ was 447 ± 63.8 μS/cm^2^ (n = 11) versus 537 ± 219 μS/cm^2^ (n = 8). The DIDS-sensitive G_out_ was 312 ± 58.5 μS/cm^2^ (n = 12) versus 322 ± 131 μS/cm^2^ (n = 7), in the absence and presence of NDPK-B 36–54, respectively. The DIDS-sensitive G_in_ was 147 ± 56.6 μS/cm^2^ (n = 12) versus 67.4 ± 43.0 μS/cm^2^ (n = 7).

To confirm that NDPK-B effect on CFTR was not confined to our chosen airway cell line, we analysed the impact of peptide NDPK-B 36–54 or NDPK-A 36–54 on chloride conductance using short-circuit current measurements (SCC) in gut biopsies [49] (n = 3). ACh induced a transient increase in SCC in the control measurements (ΔSCC, +32.7 ± 3.71 μA/cm^2^) (Fig 8c). However, in the presence of peptide NDPK-B 36–54 (100 μM), ACh failed to induce an increase in SCC (ΔSCC, -1.7 ± 1.64 μA/cm^2^) (Fig 8c). On the other hand, peptide NDPK-A 36–54 (100 μM) was without effect (ΔSCC, +27.6 ± 2.93 μA/cm^2^). The viability of the tissue pre/post-treatment was confirmed using sodium linked glucose (10 mM) absorption (ΔSCC +16.1 ± 1.6 μA/cm^2^ and +21 ± 1.9 μA/cm^2^, respectively). Thus, peptide NDPK-B 36–54 (100 μM) inhibited FSK-dependent CFTR and ORCC-mediated currents in 16HBE14o- cells as well as ACh-induced SCC measured across a sheet of intestinal biopsy.

## Discussion

This study demonstrates that NDPK-B forms a cAMP/PKA-dependent complex with CFTR at the apical membrane and is important for regulation of CFTR and ORCC channels by cAMP/PKA. We propose that the cAMP/PKA-induced NDPK-B interaction with CFTR occurs through a distinct domain within NDPK-B straddling serine 44. Second messenger cAMP regulates many signalling events that control numerous processes in airway epithelia including CFTR-dependent chloride transport [42]. In non-CF epithelia, phosphorylation of CFTR by PKA [61] is the major recognized intracellular signalling mechanism for activation of CFTR-dependent chloride flux. The inhibition of the ACh-dependent SCC by the peptide NDPK-B 36–54 in human gut biopsies provides evidence for a wider relevance of NDPK-B to epithelial function and suggests that the NDPK-B/CFTR complex is likely to be relevant towards ion transport *in vivo* across a range of epithelia given the universal expression of these isoforms of NDPK. Association of NDPK-B with the CFTR nucleotide binding domain (NBDs) is interesting because NDPKs play an important role in local provision of nucleotides to many cellular processes and suggests NDPK-B could be important for NBD function. The two NBDs dimerise to drive ATP hydrolysis and thereby regulate the opening and closing of the CFTR Cl- channel. Activation of CFTR function requires a combination of phosphorylation, ATP binding and hydrolysis and one study suggests that cAMP binding may also occur [62]. A key unexplained aspect of CFTR function is the discrepancy between the slow rates of ATP hydrolysis observed with purified CFTR and the fast gating kinetics of channel opening [63–65]. We speculate that, as for many aspects of NDPK function (G proteins, ion channel and dynamin regulation), the fast turnover of NDPK (1000 per sec) could supply the missing link between the biochemistry and the electrophysiology.

The precise mechanism by which PKA regulates the NDPK-B/CFTR complex is currently unclear but likely involves protein phosphorylation, since catalytic inhibitors of PKA disrupt both translocation and co-immunoprecipitation of NDPK-B with CFTR. CFTR is a well-defined PKA substrate and possesses several physiologically relevant PKA target sites within its regulatory domain [61]. Previous studies have demonstrated that apart from the autophosphorylation of NDPK on histidine 118, phosphorylation also occurs on serine residues (Ser-44 and Ser-120/122, near to the catalytic H118) [43, 66]. However, the significance of NDPK Ser-44 phosphorylation remains uncertain. Ser-44 exists on the hexamer surface close to negatively charged residues 45 and 46. Since the initial recognition of NDPK phosphorylation on Ser-44, the number of NDPK isoforms known has increased and interestingly, sequence analysis predicts a putative PKA target site at Ser-44 which is unique to NDPK-B and the testis specific isoform [67], NM23-H5. Paradoxically, previous studies *in vitro* show that NDPK serine phosphorylation is inhibited by cyclic AMP (with a K_i_ of 1 mM) [43, 44]. At first glance the physiological significance is unclear since the Ki was very high in the (mM) range but given that NDPK is involved in energy charge regulation, it remains possible that a very high local concentration of cyclic AMP in the vicinity of CFTR could remain relevant given that we find (non-hydrolysable) cAMP can act as a bridge to enhance the binding of CFTR to NDPK *in vitro*. Indeed it has been proposed that cAMP efflux pumps can co-localise with CFTR [68, 69] making such a scenario plausible should such efflux be inhibited.

Disruption of the NDPK-B/CFTR complex by peptide NDPK-B 36–54 demonstrates that the region of diversity between NDPK-A & B corresponding to amino acids 36–69 encompassing the β-sheet of β2 and the α-helix α_A_, and which possesses an unusual leucine cluster and an ideal structural surface location [70], is important for the cAMP/PKA-dependent interaction with CFTR. In functional studies, the peptide NDPK-B 36-54-induced inhibition of CFTR chloride conductance demonstrates that activation of CFTR-mediated currents by PKA is dependent on NDPK-B forming a complex with CFTR. Given that NDPK-B interaction with CFTR is cAMP/PKA-dependent, our application of peptide NDPK 36–54 to CFTR functional analysis provides a novel tool to distinguish between effect of PKA-mediated CFTR phosphorylation and the impact of NDPK-B/CFTR complex formation on CFTR function. In contrast, peptide NDPK-A 36–54 neither disrupts the NDPK-B/CFTR complex nor inhibits cAMP/PKA-dependent CFTR function providing both an ideal control and excluding non-specific effects of a high peptide concentration. These data also suggest that NDPK-A may interact with CFTR through a different mechanism.

Inhibition of CFTR function by peptide NDPK-B 36–54, despite PKA activation, also highlights the fact that, in addition to CFTR phosphorylation, other cellular processes are required for CFTR activation. However, this is in contradiction to O’Riordan *et al*, who found that PKA alone regulates highly purified CFTR reconstituted into planer lipid bi-layers [71]. Our data suggest that although PKA undoubtedly activates CFTR directly in planer lipid bilayers, regulation of function *in vivo* is a more complex process. For example, increasing evidence shows that a number of proteins, including syntaxin 1A/syntaxin 8/AMPKα, exist in complex with CFTR under basal conditions and inhibit CFTR function. The impact of PKA on CFTR function in lipid bilayers, in the presence of these naturally occurring inhibitory complexes is yet to be analysed.

It has been repeatedly observed that CFTR interacts with and regulates a number of other ion channels, including ORCC [72], ENaC [73] and ROMK [74]. Several possible mechanisms have been proposed for these interactions, including changes in intracellular Cl^-^ concentrations, protein-protein interactions and a role of scaffolding proteins such as NHERF [75–77]. We measured whole cell cAMP-activated DIDS-sensitive (ORCC) and CFTR Cl^-^ currents (using DIDS and CFTR_ihn172_). Incubation of the cells with peptide NDPK-B 36–54, decreased both CFTR and ORCC current magnitude indicating that both ORCC and CFTR are regulated by the interaction with NDPK-B. As ORCC are also regulated by CFTR, the interaction with NDPK-B may control CFTR-mediated regulation of other ion channels. Alternatively, NDPK-B may target ORCC independently of CFTR.

It is as yet unclear whether NDPK-B also binds to other CFTR domains (particularly extracellular domains, since several reports show NDPK-B as well as NDPK-A are released extracellularly [78]). The interaction described herein between NDPK-B and NBD1 and its disruption by NDPK-B 36–54 peptide strongly suggests that NDPK-B, following its cAMP/PKA-dependent translocation to the plasma membrane, binds to CFTR via NBD1 domain. The mechanism of cellular internalization of the NDPK-B 36–54 peptide is at present unclear. However, recent reports show several types of peptides penetrate cells and traverse the plasma membrane by various mechanisms including endocytosis and energy independent pathways [79]. In lower organisms such as flies, worms, sponges and amoebae, it is now clear that NDPKs control the balance between the different forms of cell nutrient uptake [21] (for example macro- versus micro-pinocytosis in Amoebae) which has recently been linked to cell growth [20]. Thus external peptide uptake might occur by interacting with the machinery governing nutrient uptake and endocytosis.

In conclusion, our data identifies NDPK-B as a new modulator of the cAMP/PKA-dependent ORCC and CFTR function in epithelia. Further understanding of NDPK-B regulation should uncover novel pathways required for epithelial cell secretion and function. Given the role of NDPK in accelerating nucleotide turnover (thus substantially increasing the activity of proteins such as dynamin), NDPK could enhance the turnover of CFTR-dependent nucleotide hydrolysis and further work will determine whether the discrepancy between fast gating and slow ATP-ase activity of CFTR can be explained through NDPK-B’s augmentation and interaction with CFTR. Recently, Amaral and Balch have set out a route map to understand the pleiotropy in CF disease pathogenesis as a model of defective proteostasis [80]. Their review does not focus on mechanism whereby a barely imperceptible 1 in 1480 amino acid deletion in CFTR (the one) can lead to so many changes across networks. Our data on NDPK, AMPK and CFTR could provide a new hypothesis to explain some of CF pleiotropy.

## Supporting Information

S1 File Images of whole western blots from which cut-outs in Figs 3–5 were made. (PDF)
