# Supplementary material for: Role of Interaction and Nucleoside Diphosphate Kinase B in Regulation of the Cystic Fibrosis Transmembrane Conductance Regulator Function by cAMP-Dependent Protein Kinase A
Source: PLoS One. 2016 Mar 7;11(3):e0149097. doi: 10.1371/journal.pone.0149097 (PMC4780765; doi:10.1371/journal.pone.0149097)
Supplement: S1 File — (PDF) [file pone.0149097.s001.pdf]

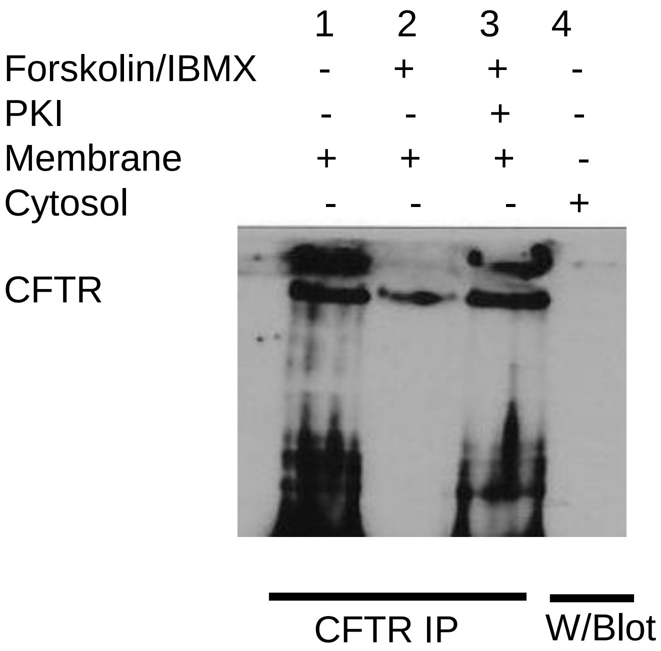

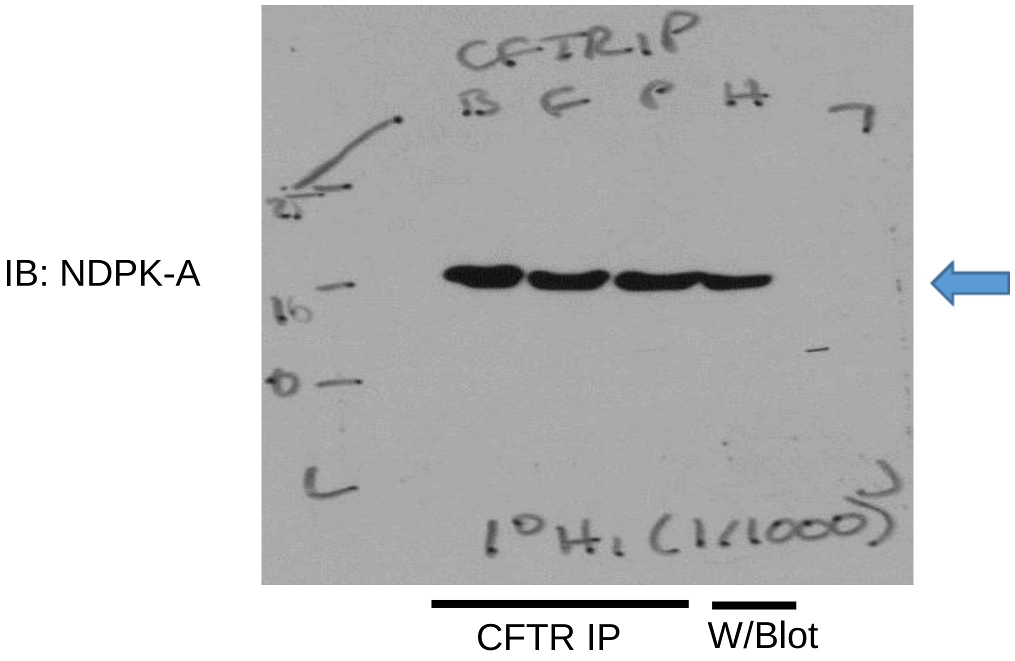

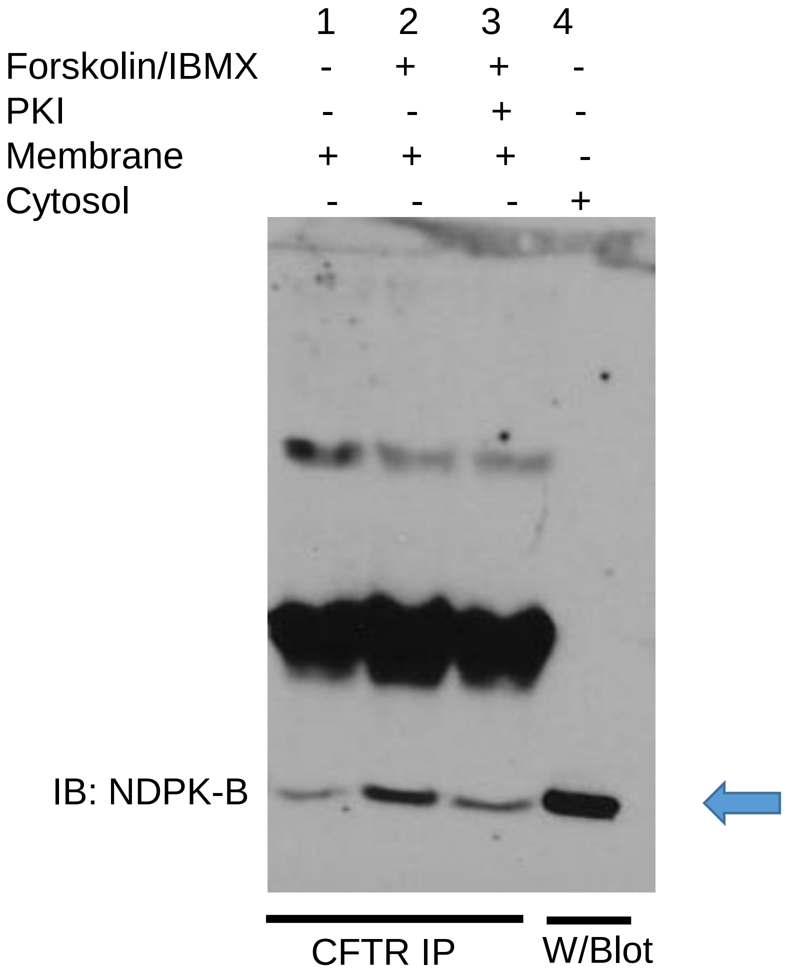

Figure 3a

Images of whole blots from which cut-outs (arrows) in figure 3a were made

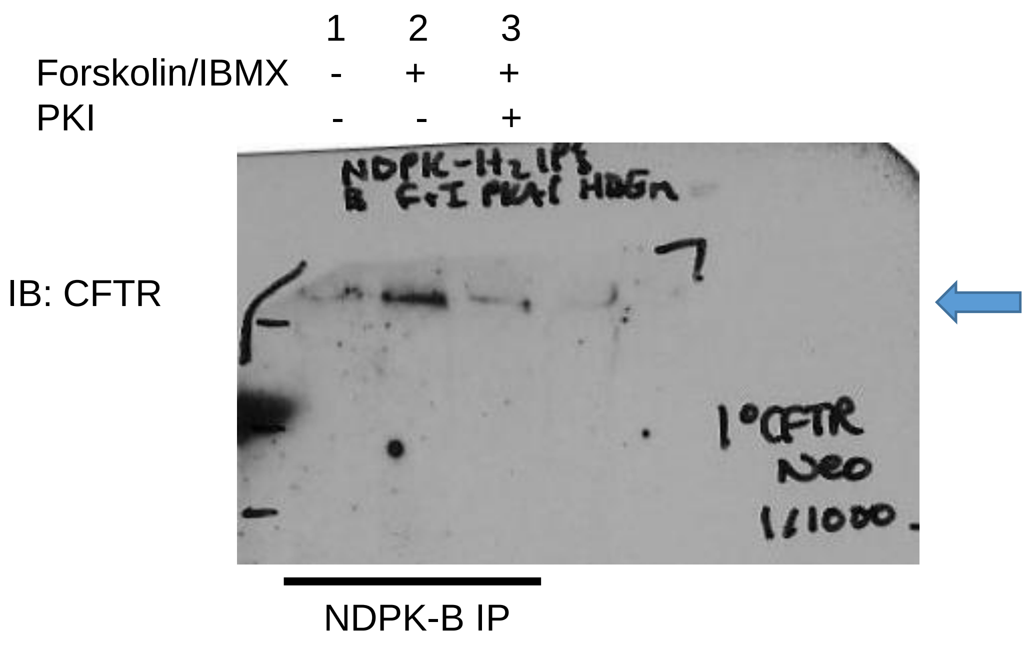

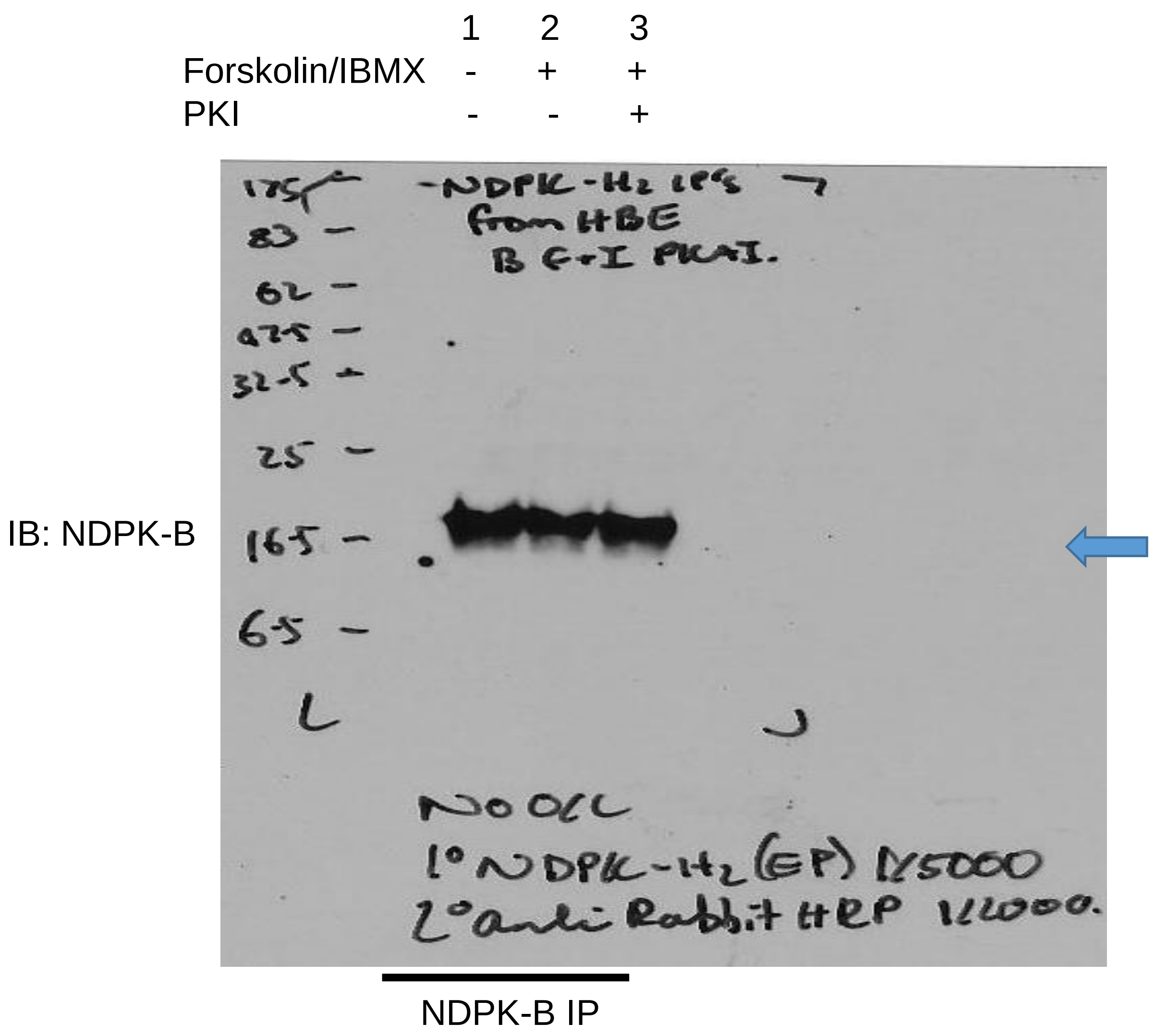

Figure 3c

Images of whole blots from which cut-outs (arrows) in figure 3c were made

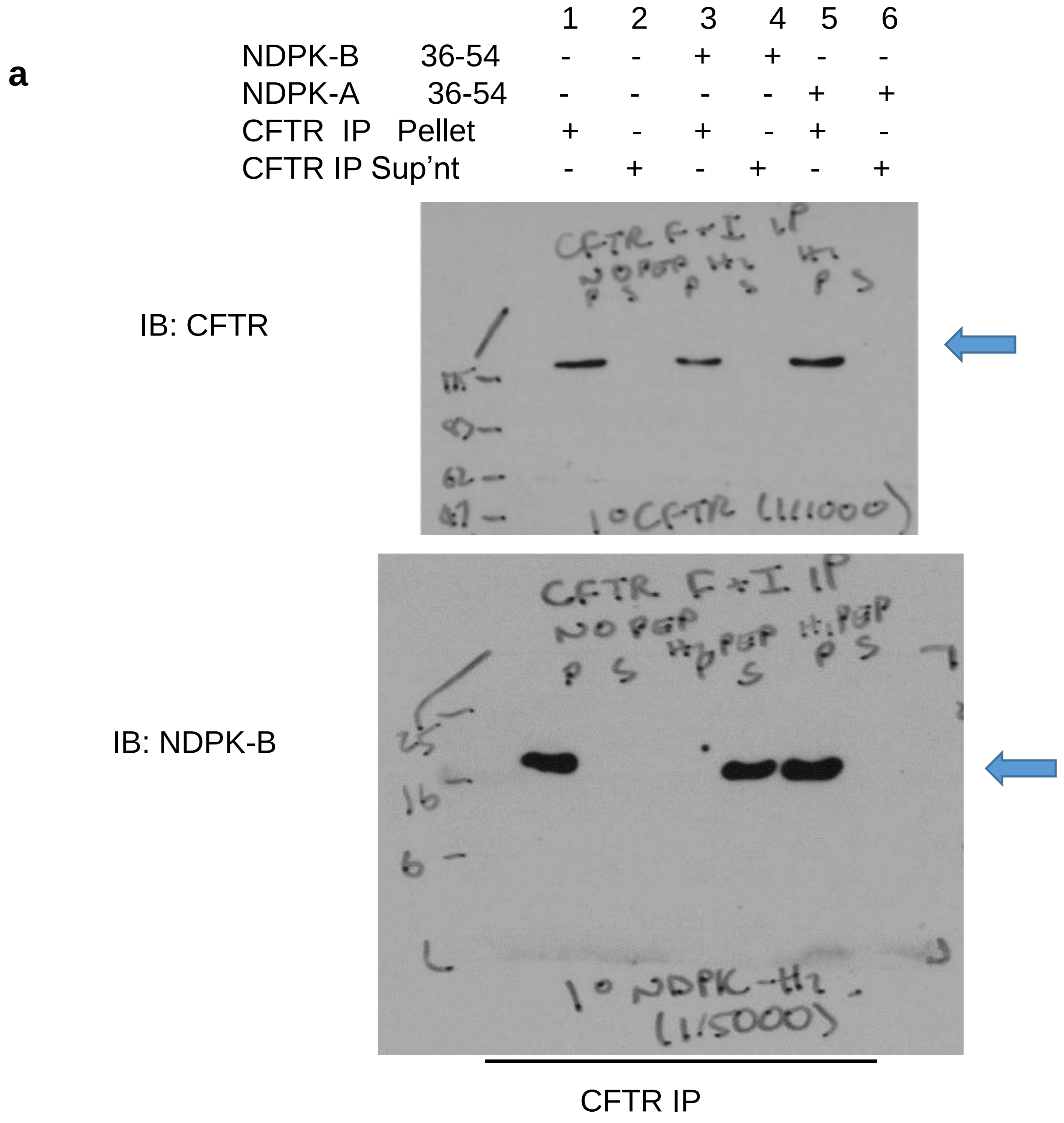

Figure 4a

Images of whole blots from which cut-outs (arrows) in figure 4a were made

b

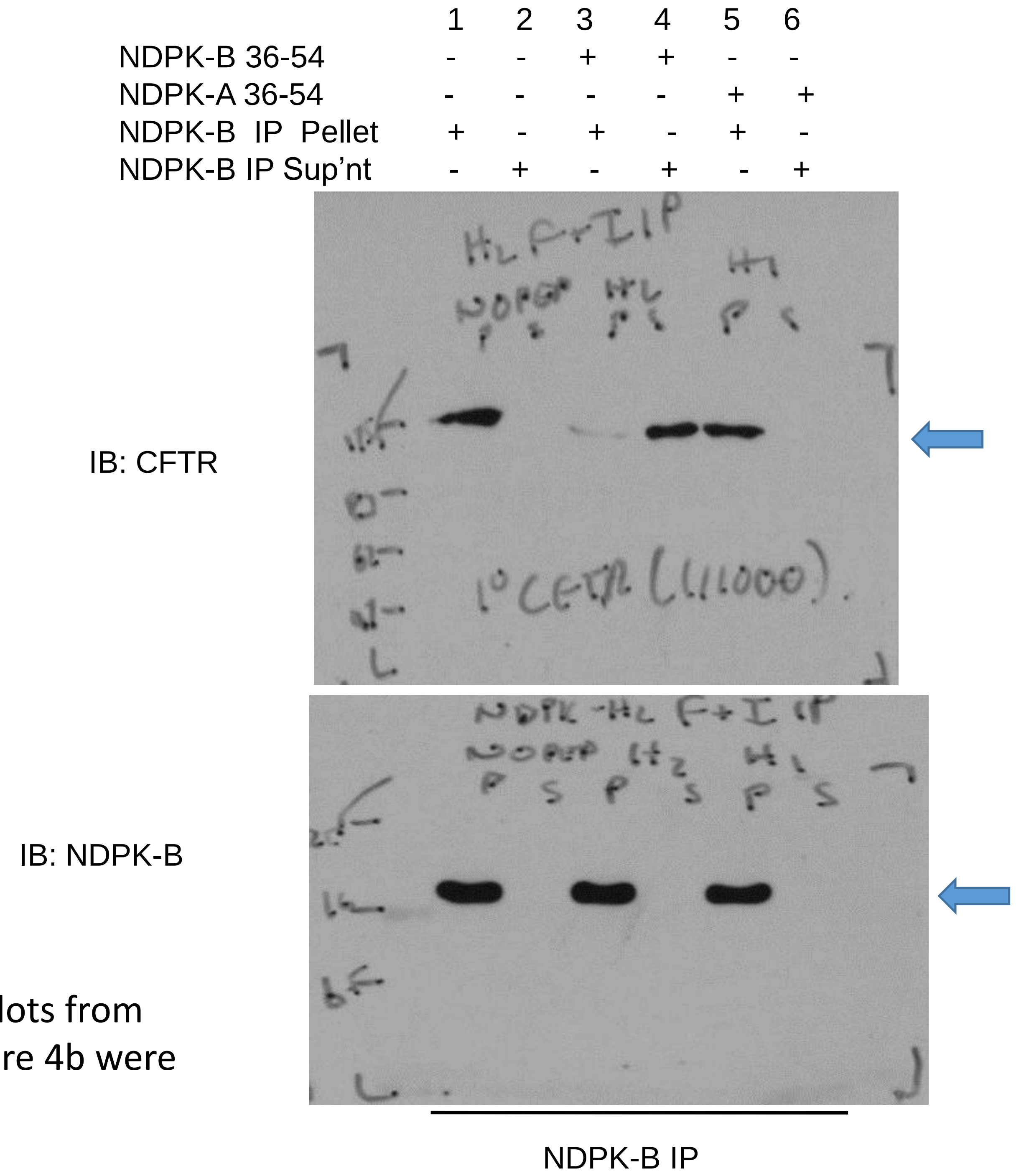

Figure 4b Images of whole blots from which cut-outs (arrows) in figure 4b were made

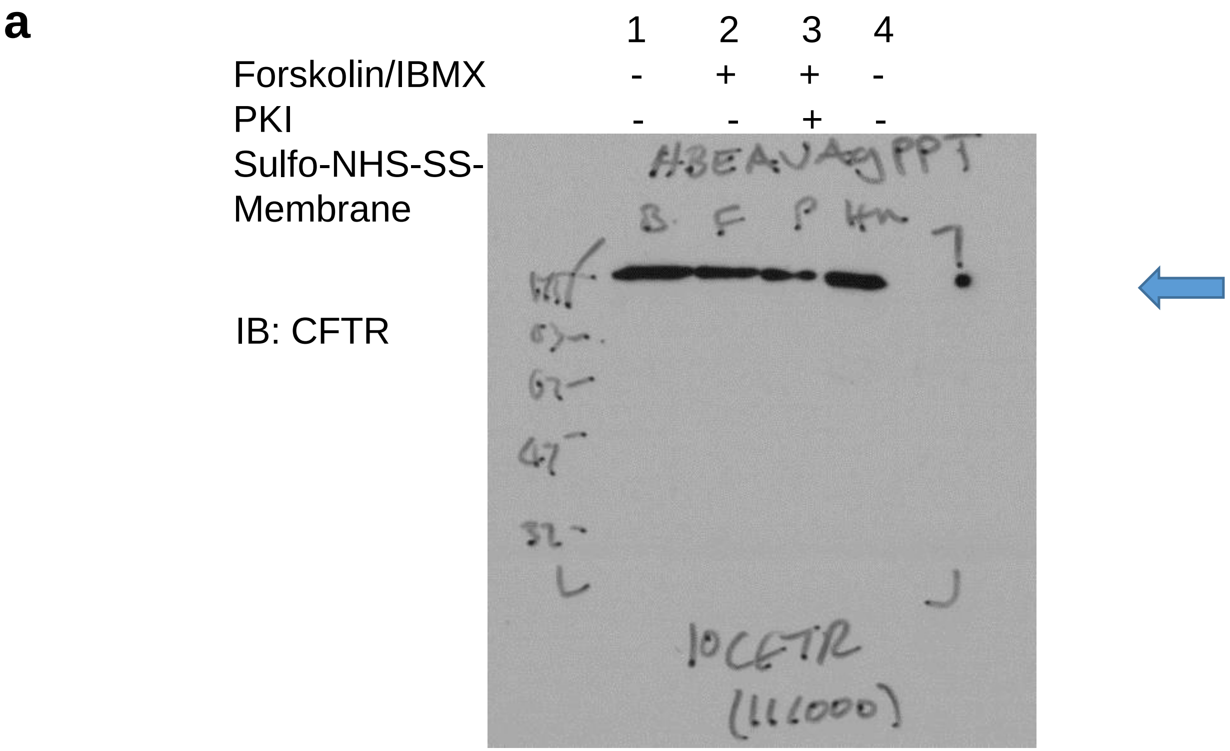

Figure 5a - Image of whole blots from which cut-out (arrows) in figure 5a were made

Figure 5b - Images of whole blots from which cut-outs (arrows) in figure 5b were made

	1	2	3	4
Forskolin/IBMX	-	+	+	-
PKI	-	-	+	-
Sulfo-NHS-SS-Biotin	+	+	+	-
Cytosol	-	-	-	+

IB: CFTR

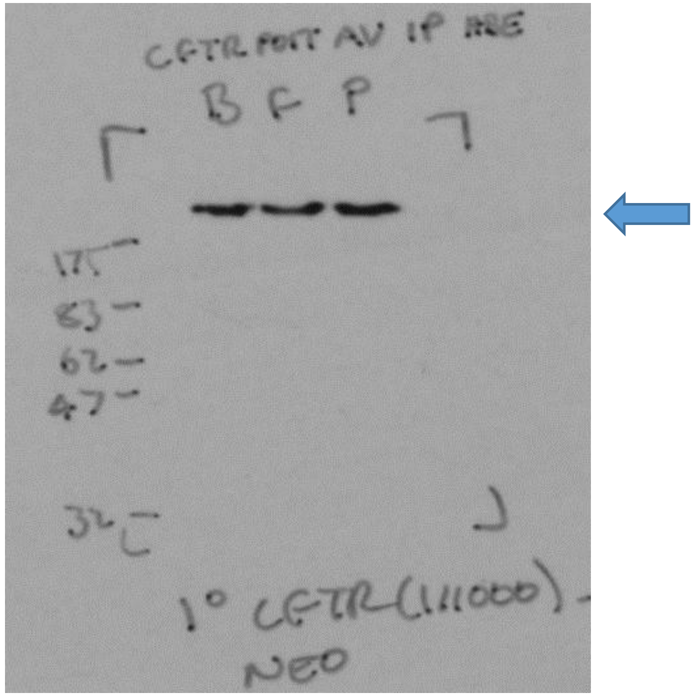

CFTR IP (Post-avidin agarose precipitate)

W/Blot

	1	2	3	4
Forskolin/IBMX	-	+	+	-
PKI	-	-	+	-
Sulfo-NHS-SS-Biotin	+	+	+	-
Cytosol	-	-	-	+

IB: NDPK-B

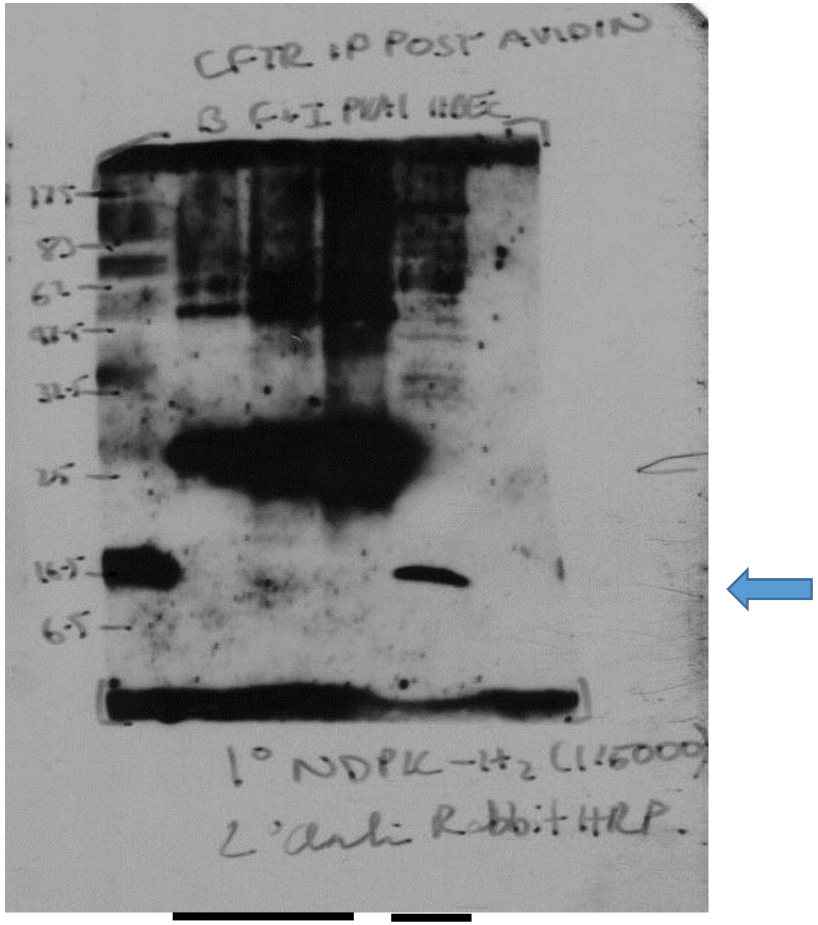

CFTR IP (Post-avidin agarose precipitate)

W/Blot

Figure 5c - Images of whole blots from which cut-outs (arrows) in figure 5c were made

	1	2	3	4
Forskolin/IBMX	-	+	+	-
PKI	-	-	+	-
Sulfo-NHS-SS-Biotin	+	+	+	-
Membrane	-	-	-	+

IB:CFTR

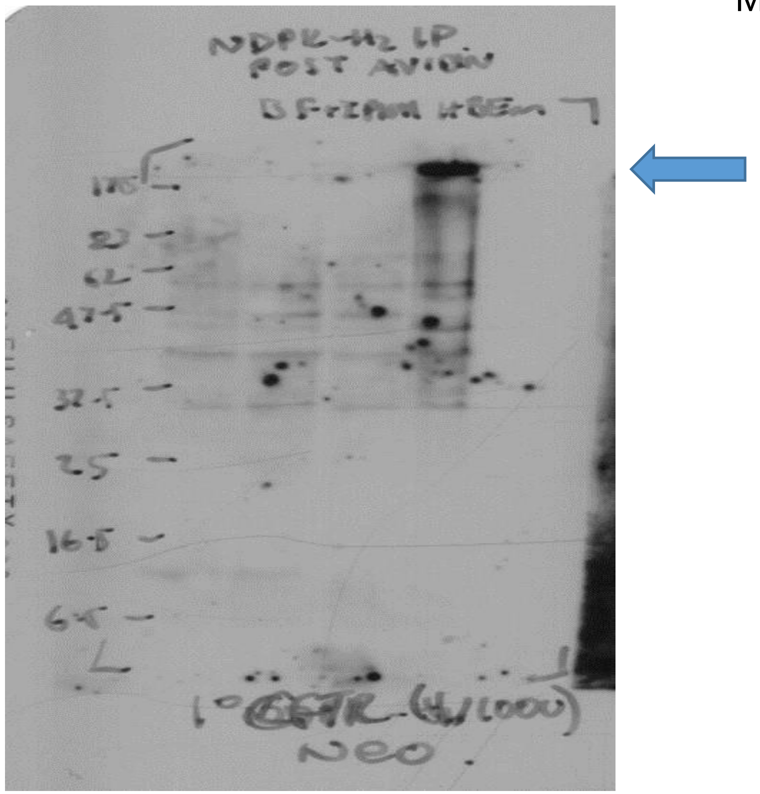

NDPK-B IP (Post-avidin agarose precipitate) W/Blot

	1	2	3	4
Forskolin/IBMX	-	+	+	-
PKI	-	-	+	-
Sulfo-NHS-SS-Biotin	+	+	+	-
Membrane	-	-	-	+

IB: NDPK-B

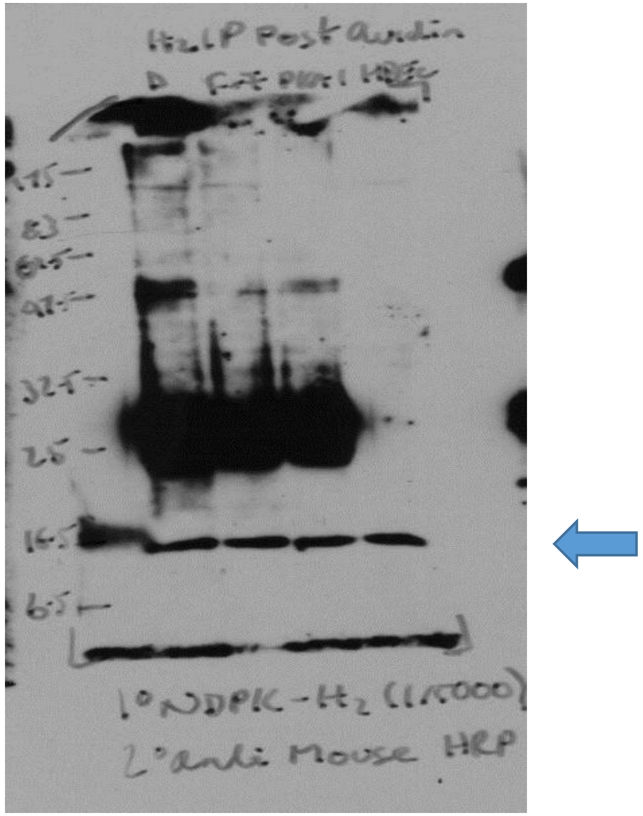

NDPK-B IP (Post-avidin agarose precipitate) W/Blot
